# Alcohol use and dementia: a systematic scoping review

**DOI:** 10.1186/s13195-018-0453-0

**Published:** 2019-01-05

**Authors:** Jürgen Rehm, Omer S. M. Hasan, Sandra E. Black, Kevin D. Shield, Michaël Schwarzinger

**Affiliations:** 1 Institute for Mental Health Policy Research, CAMH, 33 Russell Street, Toronto, Ontario M5S 2S1 Canada; 2 Dalla Lana School of Public Health, University of Toronto, 27 King’s College Circle, Toronto, M5S 1A1 Ontario Canada; 3 Campbell Family Mental Health Research Institute, CAMH, 250 College Street, Toronto, M5T 1R8 Ontario Canada; 4 Institute of Medical Science, University of Toronto, Medical Sciences Building, 1 King’s College Circle, Toronto, M5S 1A8 Ontario Canada; 5 Department of Psychiatry, University of Toronto, 250 College Street, Toronto, M5T 1R8 Ontario Canada; 6 Institute for Clinical Psychology and Psychotherapy, Technische Universität Dresden, Chemnitzer Str. 46, Dresden, 01187 Germany; 7 Department of Medicine (Neurology), Sunnybrook Health Sciences Centre and University of Toronto, 2075 Bayview Avenue, Toronto, M4N 3M5 Ontario Canada; 8 Hurvitz Brain Sciences Research Program, Sunnybrook Research Institute, Toronto, M4N 3M5 Ontario Canada; 9 Translational Health Economics Network (THEN), 39 quai de Valmy, Paris, 75010 Paris France

**Keywords:** Dementia, Alcohol, Risk, Systematic review, Alzheimer’s disease, Vascular dementia, Brain function, Brain volumetrics, Cognition

## Abstract

**Background:**

Alcohol use has been identified as a risk factor for dementia and cognitive decline. However, some patterns of drinking have been associated with beneficial effects.

**Methods and Results:**

To clarify the relationship between alcohol use and dementia, we conducted a scoping review based on a systematic search of systematic reviews published from January 2000 to October 2017 by using Medline, Embase, and PsycINFO. Overall, 28 systematic reviews were identified: 20 on the associations between the level of alcohol use and the incidence of cognitive impairment/dementia, six on the associations between dimensions of alcohol use and specific brain functions, and two on induced dementias. Although causality could not be established, light to moderate alcohol use in middle to late adulthood was associated with a decreased risk of cognitive impairment and dementia. Heavy alcohol use was associated with changes in brain structures, cognitive impairments, and an increased risk of all types of dementia.

**Conclusion:**

Reducing heavy alcohol use may be an effective dementia prevention strategy.

**Electronic supplementary material:**

The online version of this article (10.1186/s13195-018-0453-0) contains supplementary material, which is available to authorized users.

## Background

Dementia is a clinical syndrome characterized by a progressive deterioration in cognitive ability and the capacity for independent living and functioning [1]. Dementia affects memory, thinking, behavior, and the ability to perform everyday activities [2], and is a leading cause of disability in older individuals [3]. Globally, dementia affects 5 to 7% of people 60 years of age or older [4]. Furthermore, the number of people with dementia globally is projected to nearly triple, from about 50 million in 2015 to 130 to 150 million in 2050 [5, 6] and this is due primarily to the epidemiological transition of the world’s population to older individuals [7], especially in lower- and middle-income countries [6].

Given the current and projected numbers of people with dementia and the associated disability, dementia is considered a major public health priority [2]. There is a potential for intervention and prevention by targeting the risk factors involved in the pathophysiological mechanisms causing dementia, such as subcortical ischemic vascular disease, amyloid angiopathy, and cortical infarction [8, 9]. These interventions and prevention strategies may be dependent on the type of dementia, and Alzheimer’s disease (AD) is the most common, followed by vascular dementia and rarer types of dementia ((including mixed types of dementia) [1]. The 2017 Lancet Commission recommended that researchers and policy makers be “ambitious about prevention” [1]; however, there was no mention of harmful alcohol use as a potential preventative target. Although there is considerable evidence of the neurotoxicity of alcohol on the brain [10–12], the absence of alcohol use as a potential risk factor for dementia may be due, in part, to seemingly conflicting evidence from epidemiological studies.

Recent evidence from a large-scale retrospective cohort of more than 30 million French hospital patients suggests that alcohol use may play a large role in the development of early-onset dementia [13]. Specifically, among people 64 years of age and younger, the majority of dementia cases either were classified as alcohol-related or were observed in patients in whom an alcohol use disorder (AUD) had been previously diagnosed [13]. Furthermore, a prior diagnosis of an AUD was found to be significantly associated with dementia across all age and subtype categories, and observed relative risk (RR) of dementia exceeded the RRs of all other modifiable risk factors [4, 14].

Given the impact of alcohol on dementia, our study aimed to perform a systematic scoping review of alcohol and dementia research [14, 15] to address the following questions:

- What topics were discussed with respect to alcohol use and dementia? Considering theoretical distinctions in prior research regarding dimensions of alcohol, we searched specifically for average volume of alcohol consumption and patterns of heavy episodic (binge) drinking [16]. We included AUDs as an exposure indicator.
- Which aspects of the relationship between the onset of dementia and prior alcohol use have been systematically quantified, and what are the results of these analyses?
- What are the results of systematic searches that do not directly quantify the association between the onset of dementia and prior alcohol use?
- Does the relationship vary between different kinds of outcomes (types of dementia or cognitive impairment more generally)? (See also the inclusion/exclusion criteria.)
- What methodological challenges and limitations exist in assessing the relationship between the onset of dementia and prior alcohol use?

## Methods

### Scope of the systematic search

Following the PRISMA (Preferred Reporting Items for Systematic Reviews and Meta-Analyses) guidelines [17], a systematic search was performed by using OVID to identify all systematic reviews published from January 2000 to October 2017 on Medline, Embase, and PsycINFO and by using a combination of keywords and Medical Subject Headings (MeSH) terms related to alcohol use, dementia, AD, brain function, memory, and cognitive health. Additional file 1: Tables S1 to S3 in the Additional file 1 outline the exact search strategy used for each database; a PRISMA checklist is also provided in the Additional file 1. The World Alzheimer Reports were additionally used to identify potential systematic reviews [18]. A systematic search of grey literature was performed via Google but provided no contributions which fulfilled our inclusion criteria (Additional file 1: Table S5 in the Additional file 1). It is highly unlikely that systematic reviews and meta-analyses would not be published in scientific journals [19].

### Inclusion and exclusion criteria

Reviews or meta-analyses were included if they described the systematic search process with listed databases and search terms. Narrative reviews without an explicit search strategy were excluded. In addition, included studies were restricted to systematic reviews that assessed the relationship between alcohol use and cognitive health, dementia, AD, vascular and other dementias, brain function, or memory. Systematic reviews on the association between alcohol use and brain structures were also included. Studies were included if they were published in 2000 or later in order to include only reviews which were undertaken using methodological standards similar to those used today; however, this does not mean that the original studies underlying these reviews were restricted to 2000 or later (for example, [20]) (Additional file 1).

### Additional searches/sensitivity analysis

We updated our search in March 2018. In June 2018, we added a search of the same databases focussing on the Wernicke–Korsakoff syndrome and including terms for “alcohol-related brain damage” or “alcohol-induced disorders in the nervous system” (Additional file 1: Table S4) based on suggestions of one anonymous peer reviewer. No additional systematic reviews or meta-analyses were found via this last search.

### Extraction and processing

For each review and meta-analysis that met all inclusion criteria, we extracted all the underlying individual studies (see Additional file 1 for a complete listing). In addition, we extracted data on alcohol exposure measurements and dementia diagnoses, information about risk relations, types of studies included, age restrictions, and the conclusions of the reviews. Two researchers performed the searches and screened the results for inclusion. The review was registered under PROSPERO ([21]; CRD42017080668).

## Results

**Fig. 1:**
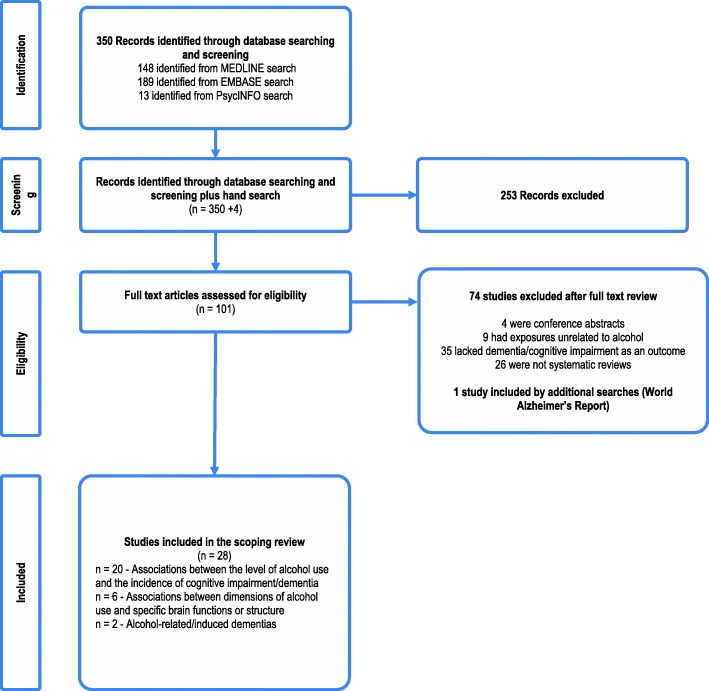
Summary of the systematic searches and the processing of information

Of the 350 results from the original search, a total of 28 systematic reviews, most of which were published after 2010 [11, 20, 22–47], met all inclusion criteria. Figure 1 outlines the results of the systematic searches.

### Research topics

The following major research topics of the published systematic reviews were identified as follows:

- the associations between the volume of alcohol consumed and the incidence of cognitive impairment/dementia, which was by far the most frequent topic of reviews (Table 1)
- the associations between the volume and patterns of alcohol use and specific brain structures and functions [29, 35, 36, 38, 39, 43]
- alcohol-related and -induced dementias [11, 22].

**Table 1:** Systematic reviews on the associations between alcohol use and the incidence of cognitive impairment or dementia, including dose-response studies

Reference	Year	Endpoint (measurement)	Major findings	Remarks: underlying studies and ages included
Hersi et al. [23]	2017	Onset and progression of AD *AD had to be determined using neuropathologic examination or by diagnosis with standardized instruments.*	Light to moderate alcohol use was associated with a decreased risk of AD onset. Heavy and daily use was associated with an increased risk. No evidence was found of the association between alcohol use and progression of AD.	Qualitative review based on seven moderate-quality systematic reviews on the association between alcohol use and risk of AD and two primary studies (see Additional file 1 for details). No age restriction as part of the inclusion/exclusion criteria.
Xu et al. [24]	2017	All-cause D; AD; VD were analyzed separately. *No measurement specification for outcome variables*	Non-linear association between alcohol use and all-cause dementia risk; the alcohol dose associated with a lower risk of dementia was confined to at most 12.5 g/day, and the risk hit bottom (RR 0.9) at roughly 6 g/day. Risk was elevated (about 10%) when the dose surpassed 23 drinks/week or 38 g/day.	MA based on 10 prospective (longitudinal) studies for all-cause dementia (Additional file 1). Relatively detailed dose-response relationships based on different dimensions (average level and frequency). No age restriction as part of the inclusion/exclusion criteria.
Cao et al. [25]	2016	All-cause D; AD; mild cognitive impairment *AD had to be determined using clearly stated diagnostic criteria or identified through diagnostic codes with additional confirmation*	Alcohol use (dichotomous) was not significantly related to the incidence of dementia as defined (see endpoint): RR 0.74, 95% CI 0.55–1.01.	MA based on eight prospective (longitudinal) studies (Additional file 1). Number of studies seems low given their inclusion/exclusion criteria. No age restriction as part of the inclusion/exclusion criteria.
LaFortune et al. [26]	2016	All-cause D; cognitive impairment *No measurement specification for outcome variables*	Consistent evidence demonstrating an association between alcohol abstinence and/or heavy drinking and cognitive impairment; compared with moderate alcohol intake, alcohol abstinence was associated with a higher risk of poor executive functioning and poor memory; one study reported no association with impairment of cognition or dementia.	Qualitative assessment of five longitudinal studies (see Additional file 1 for studies included). Minimum follow-up of 5 years. The populations covered included (1) mid-life adults (aged 40–64 years) and (2) adults aged 39 and younger in populations at a higher risk of health inequalities (people from disadvantaged and minority groups).
Cooper et al. [27]	2015	All-cause D; AD *Mild cognitive impairment was identified from neuropsychological tests, in the absence of dementia or significant functional impairment.*	Grade 2 evidence that heavy alcohol use was associated with conversion from any-type mild cognitive impairment to dementia and inconsistent evidence of whether light to moderate alcohol use predicts the risk of dementia.	Qualitative assessment of the role of alcohol in the course from mild cognitive impairment to D. Samples were based on either general populations (four studies) or clinical samples (three studies; all studies in the Additional file 1). No age restriction as part of the inclusion/exclusion criteria.
Ilomäki et al. [28]	2015	All-cause D, AD, indicators of cognitive decline *No measurement specification for outcome variables*	Light to moderate drinking was associated with the risk of AD (RR 0.72; 95% CI 0.61–0.86) and dementia (RR 0.74; 95% CI 0.61–0.91), whereas heavy to excessive drinking was not associated with either AD or D (RR 0.92; 95% CI 0.59–1.45; RR 1.04; 95% CI 0.69–1.56, respectively).	Systematic review of systematic reviews. Included only three systematic reviews [20, 37, 44] based on longitudinal studies and quality criteria (Additional file 1). No age restriction as part of the inclusion/exclusion criteria.
Xu et al. [30]	2015	AD *No measurement specification for outcome variables except that it had to be AD and not unspecified dementia*	Alcohol use, especially use of 1–3 drinks per day (RR 0.61 95% CI 0.54–0.68), but not heavier drinking, and alcohol use disorders showed a protective association (grade 1).	Included (longitudinal) cohort and retrospective case-control studies and conducted MA for different dimensions (ever versus never, 1–3 drinks per day versus never, and heavier use versus light or no use). Meta-analyses for 1–3 drinks per day based on five studies (Additional file 1). No age restriction as part of the inclusion/exclusion criteria. Excluded non-significant relationships.
Alzheimer’s Disease International [31]	2014	Incident all-cause D, AD, and VD *No measurement specification for outcome variables except that it had to be all-cause D, AD, and VD had to be directly measured*	Moderate drinkers (1–14 units for women and 1–21 for men) were at lower risk of AD (RR = 0.62, 95% CI 0.54–0.69) or any dementia (RR = 0.54, 95% CI 0.42–0.67) compared with abstainers. No significant difference between heavy drinkers and abstainers for either AD or D.	Based on Anstey et al. [20], Neafsey and Collins [40], and Peters et al. [44], plus an independent search for longitudinal studies which had measured and excluded cases with dementia at baseline (16 studies; Additional file 1). No age restriction as part of the inclusion/exclusion criteria.
Beydoun et al. [32]	2014	Incident and prevalent D, AD, cognitive function, and cognitive decline *Well-defined criteria for all outcomes*	Moderate alcohol use was associated with better cognitive function, a lower rate of cognitive decline, and a lower incidence/prevalence of dementia in the majority of cohort and cross-sectional studies (17 out of 30) included. Dose response was linear and curvilinear (J/U-shaped).	Based on 18 (longitudinal) cohort studies and 12 cross-sectional studies with qualitative assessment of results (Additional file 1) mainly with decline in cognitive function as the outcome variable. No age restriction as part of the inclusion/exclusion criteria.
Di Marco et al. [33]	2014	Dementia or any subtype thereof *No measurement specification for outcome variables*	Most studies found an association between mild to moderate alcohol use and a lower incidence of dementia.	Based on 13 cohort studies, population restricted to 35+ years old, which were free of dementia at baseline. Qualitative assessment of outcomes (Additional file 1).
Pei et al. [34]	2014	Prevalence or incidence of any type of D *Diagnosis of dementia and types of dementia had to be based on internationally recognized criteria*	Based on one study each, light to moderate alcohol use (<20 g pure alcohol per day for men and <16 g for women) was associated with a lower risk of dementia compared with those not drinking alcohol (OR 0.5; 95% CI 0.3–0.8). Daily alcohol use was associated with an increased risk of AD (OR 1.7; 95% CI 1.1–2.8).	Review was restricted to Chinese general populations, and only two studies were included with alcohol use as a risk factor (Additional file 1). Age restriction was for populations 60 years and older.
Piazza-Gardner et al. [37]	2013	AD *No measurement specification for outcome variables except that it had to be AD and not unspecified dementia (exact definition of each underlying study was given)*	No clear outcome: seven studies found an association between alcohol use and a decreased risk of AD, three studies found an association with an increased risk of AD (especially for heavier drinking), and nine studies reported no association between alcohol use and AD.	Review based on 19 studies of various designs (Additional file 1). Studies on single beverages or on symptoms only were excluded. No age restriction as part of the inclusion/exclusion criteria.
Neafsey and Collins [40]	2013	Impaired cognition; all-cause D *No measurement specification for outcome variables*	Between 1977 and 1997, mainly the associations of light to moderate alcohol use with cognitive function and impairment in young to middle-aged (18–50 years old) subjects were examined. Initial studies indicated an association of alcohol use with impaired cognition, but most later studies failed to confirm this, instead finding no difference in cognition between drinkers and non-drinkers. After 1998, mainly 55+-year-old subjects were assessed, overwhelmingly finding an association of moderate drinking with a reduced risk of dementia or cognitive impairment (RR 0.77; 95% CI 0.73–0.80) compared with non-drinkers. Heavy use (> 3–4 drinks/day) was associated with a higher risk of cognitive impairment/dementia.	Qualitative analyses of studies without a quantitative indicator of risk (OR; RR; hazard ratio) based on 69 articles. MA for quantitative studies (mainly after 1998) based on 74 studies (see Additional file 1 for listing). No age restriction as part of the inclusion/exclusion criteria; however, the results were interpreted based on age (see left column).
Lee et al. [41]	2010	All-cause D, cognitive function, decline, or impairment *Measurement of outcomes was part of a quality index*	Moderate alcohol use was associated with a lower risk of cognitive decline and dementia (compared with non-drinkers), but frequent and heavier use was associated with higher risks of dementia and cognitive impairment.	Prospective (longitudinal) studies in people older than 65 years of age were targeted, but others were included as well; qualitative analyses based on eight studies (Additional file 1).
Anstey et al. [20]	2009	All-cause D, AD, VD, cognitive decline *No measurement specification for outcome variables*	Pooled RRs of AD, VD, and all-cause D for light to moderate alcohol use compared with no use were 0.72 (95% CI 0.61–0.86), 0.75 (95% CI 0.57–0.98), and 0.74 (95% CI 0.61–0.91), respectively. Heavy use was not associated with an increased risk of any of the dementia categories.	MAs based on 15 prospective (longitudinal) studies (Additional file 1) with inclusion criteria that included ascertainment where at baselines there were no dementias, or some indication of cognitive status. No age restriction as part of the inclusion/exclusion criteria.
Purnell et al. [42]	2009	Incident AD *Formal clinical assessment procedure and use of standardized definitions for AD*	Four out of five studies found no association between alcohol use and the incidence of AD. One study reported an association with a decreased risk of AD, in part in interaction with apolipoprotein E ε4.	Qualitative assessment based on five prospective (longitudinal) studies (six articles; Additional file 1) limited to certain clinical assessment instruments of AD. No age restriction as part of the inclusion/exclusion criteria.
Peters et al. [44]	2008	Incident all-cause D; AD; VD; cognitive decline *No measurement specification for outcome variables*	MAs suggest that small amounts of alcohol were associated with a lower risk of dementia (RR 0.63; 95% CI 0.53–0.75) and AD (RR 0.57; 95% CI 0.44–0.74), but not of vascular dementia (RR 0.82; 95% CI 0.50–1.35) or cognitive decline (RR 0.89; 95% CI 0.67–1.17).	MAs based on 23 longitudinal studies (20 epidemiological cohort, three retrospective matched case-control studies nested in a cohort, reported in 26 publications; Additional file 1). Several MAs by type of dementia, sex, and various drinking measures. Only studies with subjects aged 65 years and older were included.
Patterson et al. [45]	2007	Incident all-cause D; AD; VD *“Standardized criteria”*	In the two studies identified, moderate wine consumption was associated with a reduced risk of all-cause D and AD.	Qualitative statement based on two (longitudinal) cohort studies in populations broadly similar to the Canadian population. No age restriction as part of the inclusion/exclusion criteria.
Weih et al. [46]	2007	AD *No measurement specification for outcome variables*	Most studies showed that heavy alcohol use increased dementia risk and moderate alcohol intake could reduce dementia (RR 0.55; low evidence level).	Based on seven prospective (longitudinal) studies (Additional file 1). No age restriction as part of the inclusion/exclusion criteria.
Reid et al. [47]	2002	All-cause D, cognitive impairment, cognition disorders *No measurement specification for outcome variables*	Ten studies found an increased risk of cognitive impairment associated with either a history of alcohol abuse, heavy use, or an average weekly consumption of more than 10 drinks when compared with individuals without a history of alcohol abuse or heavy drinking or compared with non-drinkers; 21 studies found no relationship between cognitive impairment and various alcohol measures. One study reported that consuming 2–5 drinks per day was associated with improved cognitive function in older women but not in men (compared with abstention).	Qualitative assessment based on 32 studies (Additional file 1) in populations older than 60 years of age. Special attention to alcohol measures, but no attempt to quantitatively summarize the findings as the measures were too different to quantitatively pool.

*Abbreviations*: *AD* Alzheimer’s disease, *CI* confidence interval, *D* dementia, *MA* meta-analysis, *OR* odds ratio, *RR* relative risk, *VD* vascular dementia

### Associations between alcohol use and the incidence of cognitive impairment/dementia, including dose-response studies

The systematic reviews published after 2000 which studied the associations between alcohol use and the incidence of cognitive impairment or dementia were often coupled with meta-analytic summaries, typically based on cohort studies which primarily measured the effect of other modifiable risk factors, usually measured at baseline (including alcohol use), on the hazard or risk (or both) of being diagnosed with cognitive impairment or dementia or dying (or both) from dementia. See Table 1 for a summary of these reviews.

The majority of these systematic reviews indicated that there was a statistically significant association between light to moderate alcohol use and a lower risk of (i) being diagnosed with cognitive impairment and different types of dementia and (ii) dying from dementia. However, two systematic reviews found inconsistent results [37, 42]. Furthermore, chronic heavy alcohol use (defined based on the World Health Organization/European Medicines Agency definitions [48, 49] as drinking more than 60 g of pure alcohol per day for men and more than 40 g of pure alcohol per day for women) was associated with an increased risk of being diagnosed with either cognitive impairment or dementia. There also was an association found between engaging in irregular heavy drinking and the risk of being diagnosed with either cognitive impairment or dementia [29, 35]. In several reviews [24, 31, 40, 41], the potential of an interaction between alcohol use and the presence or absence of the apolipoprotein E ε4 allele (a known risk factor for AD [50] and other types of dementia [51]) and the resulting risk of either cognitive impairment or dementia was also examined, albeit based on a limited number of studies with substantial heterogeneity (see also [52]).

The causality of the association between the volume and patterns of alcohol use and the development of cognitive impairment and dementia was assessed by Piazza-Gardner et al. [37], who determined that there was not sufficient evidence of a causal relationship between light to moderate drinking and a decreased risk of dementia. Overall, the level of evidence and the methodological quality of the reviews were judged to be only moderate (for a systematic evaluation of the reviews, see [23, 28]). With respect to the positive association between light and moderate drinking and vascular dementia, the underlying protective mechanisms of these patterns of use for cardiovascular outcomes were mentioned (that is, having a favorable impact on lipid levels by, for example, increasing high-density lipoprotein, and affecting atherosclerosis and inflammation via decreases in fibrinogen levels and inflammation markers [53–55]).

All meta-analyses (see the Additional file 1 for details) showed high levels of heterogeneity. The following limitations in assessing the relationship between alcohol use and the onset of cognitive impairment and dementia were highlighted in the systematic reviews and may explain, in part, the observed heterogeneity between studies:

- Alcohol use was always self-reported [24, 37] and in almost all studies was assessed only once, at baseline [37].
- There was a lack of standardization of alcohol use and of level of use categories across studies [28, 30, 33, 34, 37, 44]; some reviews used only broad descriptive categories (that is, light, moderate, and heavy alcohol use) which varied widely because of different standard alcoholic drink sizes across countries and differences in the categorization of alcohol use volumes and patterns ([56, 57]; see also [54]).
- There was inconsistent or no control for potential confounding variables, as different  risk factors or confounding variables (or both) were measured across cohort studies [24, 30, 33, 44]. Furthermore, interactions between alcohol and other risk factors, particularly tobacco smoking, may also exist but these interactions were not assessed [42].
- Former drinkers were usually grouped with lifetime abstainers to create a control group [20, 24, 28, 31, 44], leading to a lack of control for “sick quitters” (that is, people who quit drinking because of health problems [58, 59]). However, having conducted a meta-analysis of studies in which former drinkers were categorized separately, Neafsey and Collins still observed a statistically significant association between moderate drinking and a lower risk of cognitive impairment or dementia [40]. Also, Reid et al. explicitly included only studies which separated lifetime abstainers and former drinkers [47].
- Many studies lacked a quality assessment for various outcomes, with many different operationalizations for cognitive functioning [32], and lacked standardization for the diagnoses of different types of dementia [27, 37].
- Some studies highlighted that the selection processes used in cohort studies may lead to underestimation of the associations between alcohol use and cognitive impairment or dementia [20]. First, many cohort studies exclude heavier drinkers [31, 60]. Second, most of the studies on alcohol use and cognitive decline/dementia concerned older subjects ([40, 44]; Table 1). Therefore, people with heavier alcohol use may have been excluded from these studies as they may have been more likely to have dementia at baseline or may have died prior to or before the end of the study because of other alcohol-attributable causes of death [16, 61]. In particular, there was an observed increase in the risk of an alcohol-attributable death at lower levels of use, such as 30 g of pure alcohol per day, and risk accelerated exponentially as average use increased [62].
- Survivor bias may also be an issue because of missing dementia information [61] and this was not included in most reviews (exception [24]).
- Owing to these limitations, two systematic reviews [32, 47] refrained from conducting meta-analyses because of the lack of exposure or outcome comparability across studies or both.

### Associations between dimensions of alcohol use and specific brain functions

The systematic reviews that assessed the relationship between alcohol use and the resulting effects on brain structures and specific brain functioning assessed diverse associations. Verbaten tested the hypothesis that low to moderate drinking (about one to three standard alcoholic drinks) had beneficial effects on brain structure (through a review of seven magnetic resonance imaging (MRI) studies) and cognitive performance (through a review of six observational studies) [43]. In the MRI studies, a linear negative association was observed between the volume of alcohol consumed and brain volume and grey matter, and a positive linear association was observed between the volume of alcohol consumed and white matter volumes (in men but not in women). However, when restricted to people aged 65 years and older, low to moderate alcohol use was related to grade of white matter integrity and cognition in a curvilinear manner (that is, U-shaped). A recently published large-scale study with a follow-up at 30 years, which measured alcohol use every 5 years and involved multiple MRI images and cognitive tests, concluded that alcohol use, even at light or moderate levels, was associated with adverse brain outcomes, including hippocampal atrophy [63], thereby corroborating the general results of the systematic review by Verbaten for people under 65 years of age.

The systematic review by Montgomery et al. measured the association between heavy alcohol use in social drinkers and executive functioning [38]. The findings of the underlying studies were heterogeneous, and, when these studies were combined, no significant relationship between heavy alcohol use and executive functioning was observed; however, in a randomized control study by Montgomery et al., heavy alcohol use was significantly associated with all sub-measures of executive functioning except for memory updating [38]. Therefore, the detrimental effects of alcohol use may be mediated through a decrease in executive functioning [29, 35, 38]. Two other systematic reviews based on imaging studies [36, 39] found consistent detrimental effects of heavy alcohol use on brain structures and function. The structural effects have also been confirmed in autopsy studies [64]. Both functional and structural impacts of heavy use have been corroborated in a number of additional narrative reviews (for example, [11, 12, 65]).

### Alcohol-related and alcohol-induced dementia

Heavy alcohol use has been shown to be a contributory factor, as well as a necessary factor (where the disease would not exist in the absence of alcohol), in the development of multiple brain diseases and such use may cause alcohol-related brain damage in multiple ways [11, 12, 64]. First, ethanol and its metabolite acetaldehyde have a direct neurotoxic effect, leading to permanent structural and functional brain damage [66, 67]. Second, chronic heavy alcohol use can result in thiamine deficiency by causing inadequate nutritional thiamine intake, decreased absorption of thiamine from the gastrointestinal tract, and impaired thiamine utilization in the cells, leading to Wernicke–Korsakoff syndrome [68, 69]. Treatment with administration of thiamine reverses many of the Wernicke–Korsakoff syndrome symptoms, although in some people certain chronic neuropsychiatric consequences of a previous thiamine deficiency persist even with appropriate treatment [68, 70]. Third, heavy alcohol use is a risk factor for other conditions that can also damage the brain: hepatic encephalopathy in patients with cirrhotic liver disease [71], epilepsy [72], or head injury [73]. Fourth, heavy alcohol use is indirectly associated with vascular dementia because of its associations with cardiovascular risk factors and diseases such as high blood pressure, ischemic heart disease, cardiomyopathy, atrial fibrillation, and stroke (for overviews, see [16, 54]). The above associations have been identified as causal [16] and have been corroborated in studies of people with AUDs [74]. Finally, heavy alcohol use is associated with lower levels of education, tobacco smoking, and depression, all of which are risk factors for dementia.

## Discussion

Light to moderate alcohol use in middle to late adulthood was associated with a decreased risk of cognitive impairment and dementia in numerous observational studies; however, there were contradictory findings, and owing to a number of methodological weaknesses (listed in the Results section), causality of this association could not be established. Heavy alcohol use was associated with changes in brain structures as well as with cognitive and executive impairments in observational and imaging studies. Heavy alcohol use and AUDs were also associated with an increased risk for all types of dementia. Furthermore, an alcohol consumption threshold above which cognition would be impaired (reversibly or irreversibly) may exist but has not yet been identified.

This scoping review was limited by the large amount of heterogeneity in the operationalization of outcomes and the small degree of overlap of underlying studies between reviews (Additional file 1). This heterogeneity in outcome operationalization may have contributed to the contradictory findings with respect to light to moderate drinking mentioned above. Therefore, there is also a need for the use of standardized objective measures of dementia and cognitive decline, using current consensus criteria. More rigorous studies using newer dementia, genetic, and neuroimaging biomarkers are needed to establish clearer guidelines for frontline clinicians in an era in which dementia prevention is a public and individual health priority.

The observational epidemiological studies underlying the reviews listed in Table 1 were limited because the majority of the studies were restricted to older populations (that is, late adulthood). Further research is needed in representative younger populations with long overall follow-ups and better methodologies, such as the use of imaging techniques and standardized cognitive tests at different follow-up points, combined with multiple measures of exposure at baseline and the same follow-up points (that is, expanding on the designs of [63, 75]).

Furthermore, the majority of the observational study populations are not representative of heavy alcohol users or people with AUDs, as these individuals are often excluded by design [20]. Heavy alcohol users and people with AUDs were excluded from the sampling frames [60]), were more likely to drop out [20], and were more likely to die at younger ages [74, 76–78]. To address these limitations, future epidemiological studies on the role of heavy alcohol use and AUDs on dementia onset could be conducted in a hospital setting where individuals with such characteristics are over-represented.

The *Lancet* review by Livingston et al. [1] showed that the risks of heavy drinking and AUDs for dementia have been underestimated. The French hospital cohort study, indicating that AUDs represented the highest RR for dementia of all modifiable risk factors for dementia, determined that alcohol use needs to be taken into consideration by our health and social welfare systems [13]. Replication studies from other countries would also improve the evidence base [75].

Mendelian randomization studies might aid in assessing causality [79, 80] but, to date, the findings from such studies do not indicate a causal impact of alcohol on AD [81] or cognitive functioning/impairment [82, 83]. Some of the genetic markers used for alcohol consumption are problematic as their associations with average volume of drinking and with heavy drinking occasions in overall light drinkers point in opposite directions ([80]; see also the discussion following [84]). Furthermore, cohort studies in twins may contribute to identifying genetic variations [85].

## Conclusions

Given the lack of high-quality research on alcohol, AD, and cognitive functioning/impairment, future randomized prevention and secondary prevention trials with alcohol interventions are needed. Such studies would include genetic profiles, standardized cognition, mood and behavioral assessments, and quantification of structural and functional connectivity brain measures, which are all well established for dementia and found in the present scoping review to be underutilized. Such trials would be situated predominantly in the primary health-care system, where screening and brief interventions have been shown to reduce the heavy use of alcohol [86] and where many of the less severe AUDs can be treated [87]. Finally, as the addition of new analyses of existing and ongoing cohort studies will also be affected by the previously noted limitations, there is a need for future studies to address these limitations.

## Additional file

Additional file 1: Search strategies and studies included. (DOCX 264 kb)

## Abbreviations

AD: Alzheimer’s disease
AUD: Alcohol use disorder
MRI: Magnetic resonance imaging
PRISMA: Preferred Reporting Items for Systematic Reviews and Meta-Analyses
RR: Relative risk
