# Supplementary material for: Alcohol use and dementia: a systematic scoping review
Source: Alzheimers Res Ther. 2019 Jan 5;11:1. doi: 10.1186/s13195-018-0453-0 (PMC6320619; doi:10.1186/s13195-018-0453-0)
Supplement: Supplementary file 1 — Search strategies and studies included. (DOCX 264 kb) [file 13195_2018_453_MOESM1_ESM.docx]

# Additional file 1: search strategies and studies included

# To article: Alcohol use and dementia: a systematic scoping review

Contents

[Additional file 1: search strategies and studies included 1](#_Toc532874026)

[To article Alcohol use and dementia: a systematic scoping review 1](#_Toc532874027)

[Prisma checklist 2](#_Toc532874028)

[**Table S1:** Search strategy for Medline 5](#_Toc532874029)

[**Table S2:** Search strategy for Embase 6](#_Toc532874030)

[**Table S3**: Search strategy for PsycINFO 7](#_Toc532874031)

[**Table S4**: Special search for alcohol-induced brain damage and Wernicke-Korsakoff syndrome-related key words on June 17, 2018. 8](#_Toc532874032)

[**Table S5**: Google search for grey literature (October 2018) 9](#_Toc532874033)

[Studies included in each systematic review that assessed the relationship between alcohol consumption and dementia 10](#_Toc532874034)

[Number of citations in the above reviews: 21](#_Toc532874035)

[Reference List 27](#_Toc532874036)

## Prisma checklist

| **Section/topic** | **#** | **Checklist item** | **Reported on page #** |
| --- | --- | --- | --- |
| **TITLE** | | |  |
| Title | 1 | Identify the report as a systematic review, meta-analysis, or both. | 1 |
| **ABSTRACT** | | |  |
| Structured summary | 2 | Provide a structured summary including, as applicable: background; objectives; data sources; study eligibility criteria, participants, and interventions; study appraisal and synthesis methods; results; limitations; conclusions and implications of key findings; systematic review registration number. | 2 |
| **INTRODUCTION** | | |  |
| Rationale | 3 | Describe the rationale for the review in the context of what is already known. | 3-4 |
| Objectives | 4 | Provide an explicit statement of questions being addressed with reference to participants, interventions, comparisons, outcomes, and study design (PICOS). | 4 |
| **METHODS** | | |  |
| Protocol and registration | 5 | Indicate if a review protocol exists, if and where it can be accessed (e.g., Web address), and, if available, provide registration information including registration number. | 6 |
| Eligibility criteria | 6 | Specify study characteristics (e.g., PICOS, length of follow-up) and report characteristics (e.g., years considered, language, publication status) used as criteria for eligibility, giving rationale. | 5-6 |
| Information sources | 7 | Describe all information sources (e.g., databases with dates of coverage, contact with study authors to identify additional studies) in the search and date last searched. | 5-6 |
| Search | 8 | Present full electronic search strategy for at least one database, including any limits used, such that it could be repeated. | S1-S4 |
| Study selection | 9 | State the process for selecting studies (i.e., screening, eligibility, included in systematic review, and, if applicable, included in the meta-analysis). | 6 |
| Data collection process | 10 | Describe method of data extraction from reports (e.g., piloted forms, independently, in duplicate) and any processes for obtaining and confirming data from investigators. | 6 |
| Data items | 11 | List and define all variables for which data were sought (e.g., PICOS, funding sources) and any assumptions and simplifications made. | 5-6 |
| Risk of bias in individual studies | 12 | Describe methods used for assessing risk of bias of individual studies (including specification of whether this was done at the study or outcome level), and how this information is to be used in any data synthesis. | n/a |
| Summary measures | 13 | State the principal summary measures (e.g., risk ratio, difference in means). | n/a |
| Synthesis of results | 14 | Describe the methods of handling data and combining results of studies, if done, including measures of consistency (e.g., I^2^) for each meta-analysis. | n/a |
| Risk of bias across studies | 15 | Specify any assessment of risk of bias that may affect the cumulative evidence (e.g., publication bias, selective reporting within studies). | n/a |
| Additional analyses | 16 | Describe methods of additional analyses (e.g., sensitivity or subgroup analyses, meta-regression), if done, indicating which were pre-specified. | n/a |
| **RESULTS** | | |  |
| Study selection | 17 | Give numbers of studies screened, assessed for eligibility, and included in the review, with reasons for exclusions at each stage, ideally with a flow diagram. | 7 |
| Study characteristics | 18 | For each study, present characteristics for which data were extracted (e.g., study size, PICOS, follow-up period) and provide the citations. | 8 |
| Risk of bias within studies | 19 | Present data on risk of bias of each study and, if available, any outcome level assessment (see item 12). | n/a |
| Results of individual studies | 20 | For all outcomes considered (benefits or harms), present, for each study: (a) simple summary data for each intervention group (b) effect estimates and confidence intervals, ideally with a forest plot. | n/a |
| Synthesis of results | 21 | Present results of each meta-analysis done, including confidence intervals and measures of consistency. | n/a |
| Risk of bias across studies | 22 | Present results of any assessment of risk of bias across studies (see Item 15). | n/a |
| Additional analysis | 23 | Give results of additional analyses, if done (e.g., sensitivity or subgroup analyses, meta-regression [see Item 16]). | n/a |
| **DISCUSSION** | | |  |
| Summary of evidence | 24 | Summarize the main findings including the strength of evidence for each main outcome; consider their relevance to key groups (e.g., healthcare providers, users, and policy makers). | 14 |
| Limitations | 25 | Discuss limitations at study and outcome level (e.g., risk of bias), and at review-level (e.g., incomplete retrieval of identified research, reporting bias). | 14-15 |
| Conclusions | 26 | Provide a general interpretation of the results in the context of other evidence, and implications for future research. | 15-16 |
| **FUNDING** | | |  |
| Funding | 27 | Describe sources of funding for the systematic review and other support (e.g., supply of data); role of funders for the systematic review. | 19 |

## **Table S1:** Search strategy for Medline

| **Search number** | **Search terms** | **Search results** |
| --- | --- | --- |
| 1 | alcohol drinking.mp. or exp Alcohol Drinking/ | 65,640 |
| 2 | exp Alcoholic Beverages/ | 17,985 |
| 3 | drinking pattern.mp. | 642 |
| 4 | 1 or 2 or 3 | 79,999 |
| 5 | exp Cognition Disorders/ or exp Cognition/ | 211,854 |
| 6 | exp Memory Disorders/ or exp Memory/ | 139,927 |
| 7 | exp Dementia/ | 146,912 |
| 8 | exp Alzheimer Disease/ | 83,023 |
| 9 | 5 or 6 or 7 or 8 | 430,997 |
| 10 | systematic review.mp. | 103,404 |
| 11 | exp "Review"/ or exp Peer Review, Research/ or review.mp. | 2,939,187 |
| 12 | meta-analysis.mp. or exp Meta-Analysis/ | 140,406 |
| 13 | pooled analysis.mp. | 6,781 |
| 14 | 10 or 11 or 12 or 13 | 2,999,158 |
| 15 | 4 and 9 and 14 | 214 |
| 16 | 15 | 214 |
| 17 | limit 16 to (humans and yr="2000 -Current") | 157 |
| 18 | remove duplicates from 17 | **148** |

## **Table S2:** Search strategy for Embase

| **Search number** | **Search terms** | **Search results** |
| --- | --- | --- |
| 1 | alcohol drinking.mp. or exp drinking behavior/ | 48,060 |
| 2 | exp alcohol consumption/ | 105,700 |
| 3 | exp alcoholic beverage/ | 27,709 |
| 4 | exp alcoholism/ | 121,464 |
| 5 | 1 or 2 or 3 or 4 | 254,630 |
| 6 | exp Alzheimer disease/ | 164,326 |
| 7 | exp memory disorder/ | 68,383 |
| 8 | exp memory/ | 253,208 |
| 9 | exp dementia/ | 306,126 |
| 10 | exp cognitive defect/ | 135,350 |
| 11 | 6 or 7 or 8 or 9 or 10 | 637,247 |
| 12 | exp "systematic review"/ | 151,237 |
| 13 | meta analysis/ | 134,709 |
| 14 | 12 or 13 | 228,470 |
| 15 | 5 and 11 and 14 | 214 |
| 16 | 15 | 214 |
| 17 | limit 16 to (human and yr="2000 -Current") | 201 |
| 18 | remove duplicates from 17 | **189** |

## **Table S3**: Search strategy for PsycINFO

| **Search number** | **Search terms** | **Search results** |
| --- | --- | --- |
| 1 | exp ALCOHOLS/ | 16,993 |
| 2 | exp ALCOHOLISM/ | 29,009 |
| 3 | exp Binge Drinking/ | 1,965 |
| 4 | exp Drinking Behavior/ | 67,236 |
| 5 | exp Alcohol Drinking Patterns/ | 61,810 |
| 6 | 1 or 2 or 3 or 4 or 5 | 79,609 |
| 7 | exp COGNITION/ | 31,468 |
| 8 | exp MEMORY/ | 108,413 |
| 9 | exp Memory Disorders/ | 9,142 |
| 10 | exp DEMENTIA/ | 67,066 |
| 11 | exp Alzheimer's Disease/ | 40,812 |
| 12 | exp Cognitive Ability/ | 79,815 |
| 13 | exp Cognitive Impairment/ | 31,057 |
| 14 | 7 or 8 or 9 or 10 or 11 or 12 or 13 | 281,899 |
| 15 | systematic review.mp. | 18,562 |
| 16 | exp Meta Analysis/ | 4,055 |
| 17 | exp "LITERATURE REVIEW"/ | 22,334 |
| 18 | 15 or 16 or 17 | 44,299 |
| 19 | 6 and 14 and 18 | 39 |
| 20 | 19 | 39 |
| 21 | limit 20 to (human and yr="2000 -Current") | **13** |

## **Table S4**: Special search for alcohol-induced brain damage and Wernicke-Korsakoff syndrome-related key words on June 17, 2018.

Databases searched

1. Embase
2. Medline
3. PsycINFO

| **Search number** | **Search terms** | **Search results** |
| --- | --- | --- |
| 1 | exp Alcohol Drinking/ | 109509 |
| 2 | exp Alcoholic Intoxication/ | 25470 |
| 3 | exp binge drinking/ | 7275 |
| 4 | heavy drinking.mp. | 14784 |
| 5 | alcoholic beverages/ | 15559 |
| 6 | ((alcohol or ethanol) adj3 (drink* or consum* or abuse or mis-use or intake)).mp. | 354271 |
| 7 | 1 or 2 or 3 or 4 or 5 or 6 | 404322 |
| 8 | exp Korsakoff psychosis/ or exp Wernicke encephalopathy/ or alcohol-related brain damage.mp. or exp Alcohol-Induced Disorders, Nervous System/ | 133239 |
| 9 | exp Wernicke Korsakoff syndrome/ or wernicke korsakoff.mp. or exp beriberi/ or exp thiamine deficiency/ or exp Korsakoff Syndrome/ or exp Alcohol Amnestic Disorder/ or exp Thiamine Deficiency/ | 13619 |
| 10 | 8 or 9 | 140566 |
| 11 | exp Meta-Analysis as Topic/ or systematic review.mp. | 399448 |
| 12 | 7 and 10 and 11 | 531 |
| 13 | remove duplicates from 12 | 513 |
| 14 | limit 13 to human | **502** |

## **Table S5**: Google search for grey literature (October 2018)

| **Search number** | **Search terms** | **Search results** |
| --- | --- | --- |
| 1 | dementia |  |
| 2 | alcohol |  |
| 3 | systematic review OR meta-analysis |  |
| 4 | From 1/1/2010 |  |

## Studies included in each systematic review that assessed the relationship between alcohol consumption and dementia

Please refer to quantitative meta-analyses reported in Table 1, if only subsets of studies were included in the meta-analyses.

**Hersi M, Irvine B, Gupta P, Gomes J, Birkett N, Krewski D. Risk factors associated with the onset and progression of Alzheimer's disease: A systematic review of the evidence. Neurotoxicology. 2017;61:143-87.**

**systematic reviews:**

Anstey et al., 2009[1]

Graves et al., 1991[2]

Lee et al., 2010[3]

Patterson et al., 2007[4]

Peters et al., 2008[5]

Weih et al., 2007[6]

Williams et al., 2010[7]

**primary studies:**

Weyerer et al, 2011[8]

Zhou et al., 2011[9]

**Cheng C, Huang CL, Tsai CJ, Chou PH, Lin CC, Chang CK. Alcohol-related dementia: A systemic review of epidemiological studies. *Psychosomatics* 2017; 58(4): 331-42.**

Carlen et al., 1994 [10]

Draper et al., 2011 [11]

Harvey et al., 2003 [12]

Ikeda,1991 [13]

Oslin & Carry, 2003 [14]

Picard et al., 2011 [15]

Rains & Ditzler, 1993 [16]

Saxton et al., 2000 [17]

Woodburn & Johnstone, 1999 [18]

**Xu W, Wang H, Wan Y, et al. Alcohol consumption and dementia risk: a dose-response meta-analysis of prospective studies. *Eur J Epidemiol* 2017; 32(1): 31-42.**

Anttila, 2004 [19]

Espeland, 2005 [20]

Handing et al., 2015 [21]

Järvenpää, 2005 [22]

Langballe, 2015 [23]

Mukamal, 2003 [24]

Paganini-Hill, 2016 [25]

Ruitenberg, 2002 [26]

Truelsen, 2002 [27]

Zhou, 2014 [28]

**Cao L, Tan L, Wang HF, et al. Dietary patterns and risk of dementia: a systematic review and meta-analysis of cohort studies. *Mol Neurobiol* 2016; 53(9): 6144-54.**

Cervilla et al., 2000 [29]

Huang, 2002 [30]

Luchsinger, 2004 [31]

Mehlig, 2008 [32]

Ruitenberg, 2002 [26]

Simons, 2006 [33]

Solfrizzi, 2007 [34]

Stampfer et al., 2005 [35]

**Lafortune L, Martin S, Kelly S, et al. Behavioural risk factors in mid-life associated with successful ageing, disability, dementia and frailty in later life: a rapid systematic review. *PloS One* 2016; 11(2): e0144405.**

Anttila, 2004 [19]*

Beulens, 2007 [36]

Elwood, 2013 [37]*

Emberson, 2005 [38]

Englund, 2013 [39]

Flood, 2008 [40]

Iso, 2004 [41]

Lin, 2005 [42]

Moayyeri et al., 2009 [43]

Ostbye et al., 2002 [44]

Otani, 2003 [45]

Qiu, 2003 [46]

Sabia, 2009 [47]*

Sabia, 2011 [48]*

Stevens, 2009 [49]

Sun et al., 2011 [50]

Tabak, 2001 [51]

Virtaa et al., 2010 [52]*

Waki, 2005 [53]

Wang, 2010 [54]

Wannamethee, 2002 [55]

Wannamethee, 2003 [56]

Willcox, 2006 [57]

Xu, 2010 [58]

**Cooper C, Sommerlad A, Lyketsos CG, Livingston G. Modifiable predictors of dementia in mild cognitive impairment: a systematic review and meta-analysis. *Am J Psychiatry* 2015; 172: 323-34.**

Artero et al., 2008 [59]

Fellows et al., 2008 [60]

Li et al., 2011 [61]

Luck et al., 2014 [62]

Solfrizzi et al., 2007 [34]

Velayudhan et al., 2010 [63]

Xu et al., 2009 [64]

**Ilomäki J, Jokanovic N, Tan ECK, Lönnroos E. Alcohol consumption, dementia and cognitive decline: An overview of systematic reviews. *Curr Clin Pharmacol* 2015; 10: 204-12.**

Anstey et al., 2009 [65]

Peters et al., 2008 [5]

Piazza-Gardner et al., 2013 [66]

**Thayanukulvat C, Harding T. Binge drinking and cognitive impairment in young people. *Br J Nurs* 2015; 24(7): 401-7.**

Crego, et al., 2009 [67]

Scaife & Duka, 2009 [68]

Townshend. & Duka, 2005 [69]

**Xu W, Tan L, Wang HF, et al. Meta-analysis of modifiable risk factors for Alzheimer's disease. *J Neurol Neurosurg Psychiatry* 2015; 86(12): 1299-306.**

Brayne et al., 1998 [70]

Broe et al., 1990 [71]

Dai et al., 2006 [72]

Deng et al., 2006 [73]*

Ferini-Strambi et al., 1990 [74]

Fratiglioni et al., 1993 [75]

Garcia et al., 2010 [76]

Graves et al., 1990 [77]

Harmanci et al., 2003 [78]

Harwood et al., 1999 [79]

Hebert et al., 1992 [80]

Huang et al., 2002 [30]*

Kokmen et al., 1991 [81]

Kondo et al., 1994 [82]

Kukull et al., 1995 [83]

Leibovici et al., 1999 [84]

Li et al., 1992 [85]

Lindsay et al., 2002 [86]

Luchsinger et al., 2004 [31]*

Mendez et al., 1992 [87]

Mukamal et al., 2003 [24]

Ruitenberg et al., 2002 [26]*

Tyas et al., 2001 [88]

Wang et al., 1997 [89]

Weyerer et al., 2011 [8]*

Zhou et al., 2011 [9]

**Alzheimer's Disease International. World Alzheimer Report 2014: Dementia and Risk Reduction. London, Alzheimer’s Disease International; 2014.**

Deng et al., 2006 [73]

Gureje et al., 2011 [90]

Huang et al., 2002 [30]

Jarvenpaa et al., 2005 [22]

Lindsay et al., 2002 [86]

Luchsinger et al., 2004 [31]

Mehlig, et al, 2008 [32]

Mukamal et al., 2003 [24]

Ruitenberg et al., 2002 [26]

Simons et al., 2006 [33]

Truelsen et al., 2002 [27]

Weyerer et al., 2011 [8]

Yip et al., 2006 [91]

**Systematic reviews:**

Anstey et al., 2009 [1]

Peters et al., 2008 [5]

Neafsey et al., 2011 [92]

**Beydoun MA, Beydoun HA, Gamaldo AA, Teel A, Zonderman AB, Wang Y. Epidemiologic studies of modifiable factors associated with cognition and dementia: systematic review and meta-analysis. *BMC Public Health* 2014; 24(14): 643.**

Anttila, et al., 2004 [19]

Au Yeung et al., 2010 [93]

Bond et al., 2001 [94]

Bond et al., 2004 [95]

Bond et al., 2005 [96]

Britton et al., 2004 [97]

Broe et al., 1998 [98]

Carmelli et al., 1999 [99]

Chan et al., 2010 [100]

Corley et al., 2011 [101]

Dufouil et al., 1997 [102]

Edelstein et al., 1998 [103]

Elias et al., 1999 [104]

Ganguli et al., 2005 [105]

Gross et al., 2011 [106]

Hendrie et al., 1996 [107]

Kalmijn et al., 2002 [108]

Krahn et al., 2003 [109]

Leroi et al., 2002 [110]

Ngandu et al., 2007 [111]

Reid et al., 2006 [112]

Richards et al., 2005 [113]

Rodgers et al., 2005 [114]

Sabia et al., 2011 [48]

Solfrizzi et al., 2007 [34]

Stampfer et al., 2005 [35]

Wright et al., 2006 [115]

Xu et al., 2009 [64]

Zanjani et al., 2013 [116]

Zhou et al., 2003 [117]

Zuccala et al., 2001 [118]

**Di Marco LY, Marzo A, Muñoz-Ruiz M, et al. Modifiable lifestyle factors in dementia: a systematic review of longitudinal observational cohort studies. *J Alzheimers Dis* 2014; 42(1): 119-35.**

Anttila et al., 2004 [19]

Deng et al., 2006 [73]

Hebert et al., 1992 [80]

Huang et al., 2002 [30]

Kivipelto et al., 2008 [119]

Larrieu et al., 2004 [120]

Lindsay et al., 2002 [86]

Luchsinger et al., 2004 [31]

Mehlig et al., 2008 [32]

Orgogozo et al., 1997 [121]

Peters et al., 2009 [122]

Ruitenberg et al., 2002 [26]

Simons et al., 2006 [33]

**Pei JJ, Giron MS, Jia J, Wang HX. Dementia studies in Chinese populations. *Neurosci Bull* 2014; 30(2): 207-16**

Deng et al., 2006 [73]

Zhou et al., 2011 [9]

**Amrani L, de Backer L, Dom G. Piekdrinken op jonge leeftijd: gevolgen voor neurocognitieve functies en genderverschillen [Adolescent binge drinking: neurocognitive consequences and gender differences]. *Tijdschrift voor Psychiatrie* 2013; 55(9): 677-89.**

Crego et al., 2009 [67]

Crego et al., 2010 [123]

Goudriaan, 2007 [124]

Hartley et al., 2004 [125]

Heffernan et al., 2010 [126]

Heffernan & O’Neill, 2012 [127]

Johnson et al., 2008 [128]

Nederkoorn et al., 2009 [129]

Parada et al., 2011 [130]

Parada et al., 2012 [131]

Sanhueza et al., 2011 [132]

Scaife & Duka, 2009 [68]

Schweinsburg et al., 2010 [133]

Schweinsburg et al., 2011 [134]

Squeglia et al., 2011 [135]

Squeglia et al., 2012 [136]

Townshend & Duka, 2005 [69]

Weissenborn & Duka, 2003 [137]

Xiao et al., 2009 [138]

**Piazza-Gardner AK, Faffud TJ, Barry AE. The impact of alcohol on Alzheimer's disease: a systematic review. *Aging Ment Health* 2013; 17(2): 133-46.**

Bachman et al., 2003 [139]

Broe et al., 1998 [98]

Deng et al., 2006 [73]

Fratiglioni et al., 1993 [75]

Garcia et al., 2010 [76]

Harwood et al., 1999 [79]

Harwood et al., 2010 [140]

Hebert et al., 1992 [80]

Huang et al., 2002 [30]

Lindsay et al., 2002 [86]

Luchsinger et al., 2004 [31]

Ogunniyi et al., 2006 [141]

Rosen et al., 1993 [142]

Ruitenberg et al., 2002 [26]

Tsolaki et al., 1997 [143]

Tyas et al., 2000 [144]

Weyerer et al., 2011 [8]

Yoshitake et al., 1995 [145]

**Montgomery C, Fisk JE, Murphy PN, Ryland I, Hilton J. The effects of heavy social drinking on executive function: a systematic review and meta-analytic study of existing literature and new empirical findings. *Hum Psychopharmacol* 2012; 27(2): 187-99.**

Crego et al., 2009 [67]

Hartley et al., 2004 [125]

Heffernan et al., 2004 [146]

Nederkoorn et al., 2009 [129]

Piechatzek et al., 2009 [147]

Townshend & Duka, 2001 [148]

Townshend & Duka, 2005 [69]

**Neafsey EJ, Collins MA. Moderate alcohol consumption and cognitive risk. *Neuropsychiatr Dis Treat* 2011; 7: 465-84.**

Amaducci et al.,1986 [149]

Anttila et al.,2004 [19]

Au Yeung et al.,2011 [150]

Bachman et al., 2003 (USA) [139]

Bachman et al., 2003 (Nigeria) [139]

Barnes et al., 2009 [151]

Britton et al., 2004 [97]

Carmelli et al., 1999 [99]

Cervilla et al., 2000 [29]

Chan et al., 2010 [100]

Cherbuin et al., 2009 [152]

Commenges et al., 2000 [153]

Dai & Pruett, 2006 [154]

DeCarli et al., 2001 [155]

Deng et al.,2006 [73]

Dufouil et al.,1997 [102]

Edelstein et al.,1998 [103]

Ensrud et al., 1994 [156]

Espeland et al., 2005 [20]

French et al., 1985 [157]

Fujishima & Kiyohara, 2002 [158]

Galanis et al., 2000 [159]

Ganguli et al., 2005 [105]

Garcia et al., 2010 [76]

Guralnik & Kaplan, 1989 [160]

Gureje et al., 2011 [90]

Harwood et al., 1999 [79]

Hebert et al., 1992 [80]

Hendrie et al., 1996 [107]

Heyman et al., 1984 [161]

Hofman & van Duijn, 1990 [162]

Huang et al., 2002 [30]

Iliffe et al., 1991 [163]

Järvenpää et al., 2005 [22]

Kim et al., 2011 [164]

Kivipelto et al., 2008 [119]

Larrieu et al., 2004 [120]

Launer et al., 1996 [165]

Leibovici et al., 1999 [84]

Lindsay et al., 2002 [86]

Lobo et al., 2010 [166]

Lopes et al., 2010 [167]

Luchsinger et al., 2004 [31]

Luck et al., 2010 [168]

McGuire et al., 2007 [169]

Mehlig et al., 2008 [32]

Mukamal et al., 2006 [170]

Ogunniyi et al., 2006 [141]

Orgogozo et al., 1997 [121]

Peters et al., 2009 [122]

Rozzini & Trabucci, 2005 [171]

Ruitenberg et al., 2002 [26]

Rusanen et al., 2011 [172]

Sabia et al., 2009 [47]

Simons et al., 2006 [33]

Smith et al., 2010 [173]

Solfrizzi et al., 2007 [34]

Stampfer et al., 2005 [35]

Tanaka et al., 2002 [174]

Truelsen et al., 2002 [27]

Tsolaki et al., 1997 [143]

Tyas et al., 2000 [144]

Umegaki et al., 2008 [175]

Vincze et al., 2007 [176]

Virtaa et al., 2010 [52]

Wadley et al., 2007 [177]

Weyerer et al., 2011 [8]

Wright et al., 2006 [178]

Yaffe et al., 2009 [179]

Yen et al., 2010 [180]

Yip et al., 2006 [91]

Yoshitake et al., 1995 [145]

Zhou et al., 2003 [117]

Zuccala et al., 2001 [118]

**Lee Y, Back JH, Kim J, et al. Systematic review of health behavioral risks and cognitive health in older adults. *Int Psychogeriatr* 2010; 22(2): 174-87**

Anttila et al., 2004 [19]

Dai et al., 2006 [72]

Elias et al., 1999 [104]

Ganguli et al., 2005 [105]

Kivipelto et al., 2008 [119]

Luchsinger et al., 2004 [31]

Ngandu et al., 2007 [111]

Simons et al., 2006 [33]

**Anstey KJ, Mack HA, Cherbuin N. Alcohol consumption as a risk factor for dementia and cognitive decline: Meta-analysis of prospective studies. *Am J Geriatr Psychiatry* 2009; 17(7): 542-55.**

Deng et al., 2006 [73]

Dufouil et al., 2000 [181]

Espeland et al., 2005 [20]

Ganguli et al., 2005 [105]

Huang et al.,2002 [30]

Larrieu et al.,2004 [120]

Lindsay et al., 2002 [86]

Luchsinger et al.,2004 [31]

Mukamal et al.,2003 [24]

Ogunniyi et al.,2006 [141]

Ruitenberg et al.,2002 [26]

Shin et al., 2005 [182]

Simons et al.,2006 [33]

Yip et al.,2006 [91]

Yoshitake et al.,1995 [145]

**Purnell C, Gao S, Callahan CM, Hendrie HC. Cardiovascular risk factors and incident Alzheimer disease: a systematic review of the literature. *Alzheimer Dis Assoc Disord* 2009; 23(1): 1-10.**

Dai et al., 2006 [72]

Fujishima & Kiyohara, 2002 [158]

Luchsinger et al., 2004 [31]

Ogunniyi et al., 2006 [141]

Ruitenberg et al., 2002 [26]

Scarmeas et al., 2006 [183]

**Verbaten MN. Chronic effects of low to moderate alcohol consumption on structural and functional properties of the brain: beneficial or not? *Hum Psychopharmacol* 2009; 24(3): 199-205.**

**For Magnetic Resonance Induction studies:**

Antsey et al., 2006 [184]

De Bruin et al., 2005 [185]

Den Heijer et al., 2004 [186]

Ding et al., 2004 [187]

Kubota et al., 2001 [188]

Mukamal et al., 2001 [189]

Taki et al., 2004 [190]

**For cognitive performance studies:**

Galanis, 2000 [159]

Lindeman, 2005 [191]

Schinka et al., 2002 [192]

Stampfer et al., 2005 [35]

Wright et al., 2006 [115]

Zhang, et al., 2005 [193]

**Peters R, Peters J, Warner J, Beckett N, Bulpitt C. Alcohol, dementia and cognitive decline in the elderly: a systematic review. *Age and ageing* 2008; 37(5): 505-12.**

Anttila et al., 2004 [19]

Broe et al.,1998 [98]

Cervilla et al., 2000a [194]

Cervilla et al., 2000b [29]

Dufouil et al., 2000 [181]

Espeland et al., 2005 [20]

Fujishima et al., 2002 [158]

Ganguli et al., 2005 [105]

Hébert et al., 2000 [195]

Huang et al., 2002 [30]

Järvenpää et al., 2005 [22]

Kivipelto et al., 2001 [196]

Larrieu et al., 2004 [120]

Launer et al., 1996 [165]

Leibovici et al., 1999 [84]

Leroi et al., 2002 [110]

Lindsay et al., 2002 [86]

Luchsinger et al., 2004 [31]

Mukamal et al., 2003 [24]

Orgogozo et al., 1997 [121]

Ruitenberg et al., 2002 [26]

Simons et al., 2006 [33]

Stampfer et al., 2005 [35]

Truelsen et al., 2002 [27]

Verhaeghen et al., 2003 [197]

Yoshitake et al., 1995 [145]

**Patterson, C., Feightner, J., Garcia, A., MacKnight, C., 2007. General risk factors for dementia: a systematic evidence review. Alzheimer's Dementia 3 (4), 341–347.**

Larrieu et al., 2004 [120]

Orgogozo et al., 1997 [121]

**Weih M, Wiltfang J, Kornhuber J. Non-pharmacologic prevention of Alzheimer's disease: nutritional and life-style risk factors. *J Neural Transm (Vienna)* 2007; 114(9): 1187-97.**

Anttila et al., 2004 [19]

Lindsay et al., 2002 [86]

Luchsinger et al., 2004 [198]

Mukamal et al., 2003 [24]

Ruitenberg et al., 2002 [26]

Truelsen et al., 2002 [27]

Yoshitake et al., 1995 [145]

**Reid MC, Boutros NN, O'Connor PG, Cadariu A, Concato J. The health-related effects of alcohol use in older persons: a systematic review. *Subst Abus* 2002; 23(3): 149-64.**

Amaducci et al., 1986 [149]

Broe et al., 1990 [71]

Canadian Study of Health and Aging, 1994 [199]

Cattin et al., 1997 [200]

Colsher & Wallace, 1990 [201]

Christian et al., 1995 [202]

Dent et al., 1997 [203]

Dufouil et al., 1997 [102]

Fratiglioni et al., 1993 [75]

French et al., 1985 [157]

Geroldi et al., 1994 [204]

Goodwin et al., 1987 [205]

Gorelick et al., 1993 [206]

Graves et al., 1990 [77]

Hebert et al., 1992 [80]

Hebert et al., 1993 [207]

Hendrie et al., 1996 [107]

Iliffe et al., 1991 [163]

Lammi et al., 1989 [208]

Launer et al., 1996 [165]

Letenneur et al., 1993 [209]

Lindsay et al., 1997 [210]

Mangione et al., 1993 [211]

Mellström et al., 1981 [212]

Orgogozo et al., 1997 [121]

Rosen et al., 1993 [142]

Saunders et al., 1991 [213]

Scherr et al., 1988 [214]

Teri et al., 1990 [215]

Tsolaki et al., 1997 [143]

Ueda et al., 1992 [216]

Yoshitake et al., 1995 [145]

## Number of citations in the above reviews:

Ruitenberg, A., et al., 2002:. eleven citations

Luchsinger, J.A., et al., 2004: ten citations

Anttila, T., et al., 2004: eight citations

Huang, W. , et al., 2002: eight citations

Lindsay, J., et al., 2002: eight citations

Deng, J., et al., 2006: seven citations

Simons, L.A., et al., 2006:. seven citations

Mukamal, K.J., et al., 2003: six citations

Orgogozo, J.M., et al., 1997: six citations

Yoshitake, T., et al., 1995: six citations

Ganguli, M., et al., 2005: five citations

Hebert, L.E., et al., 1992: five citations

Larrieu, S., et al., 2004: five citations

Truelsen, T., et al., 2002: five citations

Weyerer, S., et al., 2011: five citations

Espeland, M. A., et al., 2005: four citations

Mehlig, K., et al., 2008: four citations

Solfrizzi, V., et al., 2007:. four citations

Stampfer, M.J., et al., 2005: four citations

Zhou, R., et al., 2011: four citations

Anstey, K. J., et al., 2009: two citations

Bachman, D.L., et al., 2003: three citations

Broe, G.A., et al., 1998: three citations

Cervilla, J.A., et al., 2000b: three citations

Crego, A., et al., 2009: three citations

Dai, Q., et al., 2006: three citations

Dufouil, C., et al., 1997: three citations

Fratiglioni, L., et al., 1993: three citations

Garcia, A.M., et al., 2010: three citations

Harwood, D.G., et al., 1999: three citations

Hendrie, H.C., et al., 1996: three citations

Järvenpää, T., et al., 2005: three citations

Kivipelto, M., et al., 2008: three citations

Launer, L. J., et al., 1996: three citations

Leibovici, D., et al., 1999: three citations

Townshend, J.M., & Duka, T. , 2005: three citations

Tsolaki, M., et al., 1997: three citations

Amaducci, et al., 1986: two citations

Britton, A., et al., 2004: two citations

Broe, G.A., et al., 1990: two citations

Carmelli, D., et al., 1999: two citations

Chan, K.K., et al., 2010: two citations

French, L. R., et al., 1985: two citations

Fujishima, M., & Kiyohara, Y., 2002: two citations

Galanis, D. J., et al., 2000: two citations

Graves, A.B., et al., 1990: two citations

Hartley, D.E., et al., 2004: two citations

Iliffe, S., et al., 1991: two citations

Leroi, I., et al., 2002: two citations

Letenneur, L., et al., 1993: two citations

Nederkoorn, C., et al., 2009: two citations

Ngandu, T. , et al., 2007: two citations

Peters, R., et al., 2009: two citations

Peters, R., et al., 2008: two citations

Rosen, J., et al., 1993: two citations

Sabia, S., et al., 2011:. two citations

Sabia, S., et al., 2009:. two citations

Scaife, J.C., & Duka, T. , 2009:. two citations

Tyas, S.L., et al., 2000: two citations

Virtaa, J. J., et al., 2010: two citations

Wright, C. B., et al., 2006: two citations

Xu, G., et al., 2009: two citations

Yip, A.G., et al., 2006: three citations

Zhou, H., et al., 2003: two citations

Zuccala, G., et al., 2001: two citations

Anstey, K. J., et al., 2006: one citation

Artero, S., et al., 2008: one citation

Au Yeung, S. L., et al., 2011: one citation

Au Yeung, et al., 2010: one citation

Barnes, D. E., et al., 2009: one citation

Beulens, J. W., et al., 2007: one citation

Bond, G.E., et al., 2001: one citation

Bond, G.E., et al., 2004: one citation

Bond, G.E., et al., 2005: one citation

Brayne, C., et al., 1998: one citation

Canadian Study of Health and Aging, 1994: one citation

Carlen, P.L., et al., 1994: one citation

Cattin, L., et al., 1997: one citation

Cervilla, J. A., et al., 2000a: one citation

Cherbuin, N., et al., 2009: one citation

Christian, J. C., et al., 1995: one citation

Colsher, P. L., & Wallace, R. B., 1990: one citation

Commenges, D., et al., 2000: one citation

Corley, J., et al., 2011: one citation

Crego, A., et al., 2010: one citation

Dai, Q., & Pruett, S.B., 2006: one citation

de Bruin, E. A., et al., 2005: one citation

DeCarli, C.,et al., 2001: one citation

den Heijer, T., et al., 2004: one citation

Dent, O.F., et al., 1997: one citation

Ding, J., et al., 2004: one citation

Draper, B., et al., 2011: one citation

Elwood, P., et al., 2013: one citation

Emberson, J. R.,et al., 2005: one citation

Englund, U., et al., 2013: one citation

Ensrud, K. E., et al., 1994: one citation

Fellows, L., et al., 2008: one citation

Ferini-Strambi, L., et al., 1990: one citation

Flood, A., et al., 2008: one citation

Fujishima et al., 2002: one citation

Geroldi, C., et al., 1994: one citation

Goodwin, J.S., et al., 1987: one citation

Gorelick, P.B., et al., 1993: one citation

Goudriaan, A.E., et al., 2007: one citation

Graves, A.B., et al., 1991: one citation

Gross, A.L., et al., 2011: one citation

Guralnik, J.M., & Kaplan, G.A., 1989: one citation

Gureje, O., et al., 2011: two citations

Handing, E.P., et al., 2015: one citation

Harmancini, H., et al., 2003: one citation

Harvey, R.J., et al., 2003: one citation

Harwood, D. G., et al., 2010: one citation

Hebert, L.E., et al., 1993: one citation

Hébert, R., et al., 2000: one citation

Heffernan, T., et al., 2010: one citation

Heffernan, T., & O'Neill, T., 2012: one citation

Heffernan, T.M., et al., 2004: one citation

Heyman, A., et al., 1984: one citation

Hofman, A., & van Duijn, C.M., 1990: one citation

Ikeda, H., 1991: one citation

Iso, H., et al., 2004: one citation

Jarvenpaa et al., 2005: one citation

Johnson, C.A., et al., 2008: one citation

Kalmijn, S., et al., 2002: one citation

Kim, K. W., et al., 2011: one citation

Kivipelto, M., et al., 2001: one citation

Kokmen, E., et al., 1991: one citation

Kondo, K., et al., 1994: one citation

Krahn, D., et al., 2003: one citation

Kubota, M., et al., 2001: one citation

Kukull, W. A., et al., 1995: one citation

Lammi, U.K., et al., 1989: one citation

Langballe, E. M., et al., 2015: one citation

Lee, Y., et al, 2010: one citation

Lemeshow, S., et al., 1998: one citation

Li, G., et al., 1992: one citation

Li, J., et al., 2011: one citation

Lin, Y., et al., 2005: one citation

Lindeman, R.D., et al,: one citation

Lindsay, J., et al., 1997: one citation

Lobo, E., et al., 2010: one citation

Lopes, M. A., et al., 2010: one citation

Luchsinger, J. A., & Mayeux, R., 2004: one citation

Luck, T., et al., 2010: one citation

Luck T., et al., 2014: one citation

Mangione, C. M., et al.,1993: one citation

McGuire, L.C., et al., 2007: one citation

Mellström, D., et al., 1981: one citation

Mendez, M.F., et al., 1992: one citation

Moayyeri, A., et al., 2009: one citation

Mukamal, K. J., et al., 2001: one citation

Mukamal, K.J., et al., 2006: one citation

Ogunniyi, A., et al., 2006: four citations

Oslin, D.W., & Cary, M.S., 2003: one citation

Ostbye, T., et al., 2002: one citation

Otani, T., et al., 2003: one citation

Paganini-Hill, A., et al., 2016: one citation

Parada, M., et al., 2011: one citation

Parada, M., et al., 2012: one citation

Patterson, C., et al., 2007: one citation

Piazza-Gardner, A.K., et al., 2013: one citation

Picard, C., et al., 2011: one citation

Piechatzek, M., et al., 2009: one citation

Qiu, D., et al., 2003: one citation

Rains, V.S., & Ditzler, T.F., 1993: one citation

Reid, M.C., et al., 2006: one citation

Richards, M., et al., 2005: one citation

Rodgers, B., et al., 2005: one citation

Rozzini, R., & Trabucchi, M. , 2005: one citation

Rusanen, M., et al., 2011: one citation

Sanhueza, C., et al., 2011: one citation

Saunders, P. A., et al., 1991: one citation

Saxton, J., et al., 2000: one citation

Scarmeas, N., et al., 2006: one citation

Scherr, P. A., et al., 1988: one citation

Schinka, J.A., et al., 2002: one citation

Schweinsburg, A.D., et al., 2010: one citation

Schweinsburg, A.D., et al., 2011: one citation

Shin, I. S., et al., 2005: one citation

Smith, K., et al., 2010:. one citation

Squeglia, L.M., et al., 2011: one citation

Squeglia, L.M., et al., 2012: one citation

Stampfer, M.J., et al., 2005: one citation

Stevens, R. J., et al., 2009: one citation

Sun, Q., et al., 2011: one citation

Tabak, C., et al., 2001: one citation

Taki, Y., et al., 2004: one citation

Tanaka, N., et al., 2002: one citation

Teri, L., et al., 1990: one citation

Townshend, J.M., & Duka, T. , 2001: one citation

Tyas, S.L., et al., 2001: one citation

Ueda, K., et al., 1992: one citation

Umegaki, H., et al., 2008: one citation

Velayudhan, L., et al., 2010: one citation

Verhaeghen, P., et al., 2003: one citation

Vincze, G., et al., 2007: one citation

Wadley, V. G., et al., 2007: one citation

Waki, K., et al., 2005: one citation

Wang, L., et al., 2010: one citation

Wang, P.N., et al., 1997: one citation

Wannamethee, S. G., & Shaper, A. G. , 2003: one citation

Wannamethee, S.G., & Shaper, A.G. , 2002: one citation

Weih, M., et al., 2007: one citation

Weissenborn, R., & Duka, T. , 2003: one citation

Willcox, B. J., et al., 2006: one citation

Williams J.W., et al., 2010: one citation

Woodburn, K.J., & Johnstone, E.C. , 1999: one citation

Wright, C.B., et al., 2006: one citation

Xiao, L., et al., 2009: one citation

Xu, Q., et al., 2010: one citation

Yaffe, K., et al., 2009: one citation

Yen, C. H., et al., 2010: one citation

Zanjani, F., et al., 2013: one citation

Zhang, Y., et al., 2005: one citation

Zhou, S., et al., 2014: one citation

## Reference List

1. Anstey KJ, Mack HA, Cherbuin N. Alcohol consumption as a risk factor for dementia and cognitive decline: meta-analysis of prospective studies. Am J Geriatr Psychiatry. 2009;17:542-55. doi: 10.1097/JGP.0b013e3181a2fd07.

2. Graves AB, van Duijn CM, Chandra V, Fratiglioni L, Heyman A, Jorm AF, et al. Alcohol and tobacco consumption as risk factors for Alzheimer's disease: a collaborative re-analysis of case-control studies. EURODEM Risk Factors Research Group. International Journal of Epidemiology. 1991;20:S48-S57.

3. Lee Y, Back JH, Kim J, Kim SH, Na DL, Cheong HK, et al. Systematic review of health behavioral risks and cognitive health in older adults. International Psychogeriatrics. 2010;22:174-87. doi: 10.1017/S1041610209991189.

4. Patterson C, Feightner J, Garcia A, MacKnight C. General risk factors for dementia: a systematic evidence review. Alzheimers Dement. 2007;3:341-7. doi: 10.1016/j.jalz.2007.07.001.

5. Peters R, Peters J, Warner J, Beckett N, Bulpitt C. Alcohol, dementia and cognitive decline in the elderly: a systematic review. Age and Ageing. 2008;37:505-12.

6. Weih M, Wiltfang J, Kornhuber J. Non-pharmacologic prevention of Alzheimer's disease: nutritional and life-style risk factors. J Neural Transm (Vienna). 2007;114:1187-97. doi: 10.1007/s00702-007-0704-x.

7. Williams JW, Plassman BL, Burke J, Benjamin S. Preventing Alzheimer's disease and cognitive decline. Evid Rep Technol Assess (Full Rep). 2010:1-727. <http://www.ncbi.nlm.nih.gov/pubmed/21500874>.

8. Weyerer S, Schaufele M, Wiese B, Maier W, Tebarth F, van den Bussche H, et al. Current alcohol consumption and its relationship to incident dementia: results from a 3-year follow-up study among primary care attenders aged 75 years and older. Age Ageing. 2011;40:456-63.

9. Zhou R, Deng J, Zhang M, Zhou HD, Wang YJ. Association between bone mineral density and the risk of Alzheimer's disease. Journal of Alzheimer's Disease. 2011;24.

10. Carlen PL, McAndrews MP, Weiss RT, Dongier M, Hill JM, Menzano E, et al. Alcohol-related dementia in the institutionalized elderly. Alcoholism: Clinical & Experimental Research. 1994;18:1330-4.

11. Draper B, Karmel R, Gibson D, Peut A, Anderson P. The hospital dementia services project: age differences in hospital stays for older people with and without dementia. International Psychogeriatrics. 2011;23:1649-58.

12. Harvey RJ, Skelton-Robinson M, Rossor MN. The prevalence and causes of dementia in people under the age of 65 years. Journal of neurology, neurosurgery, and psychiatry. 2003;74:1206-9.

13. Ikeda H. Clinical and epidemiological studies of alcoholic dementia. Arukoru Kenkyu Yakubutsu Izon. 1991;26:341-8.

14. Oslin DW, Cary MS. Alcohol-related dementia: validation of diagnostic criteria. American Journal of Geriatric Psychiatry. 2003;11:441-7.

15. Picard C, Pasquier F, Martinaud O, Hannequin D, Godefroy O. Early onset dementia: characteristics in a large cohort from academic memory clinics. Alzheimer disease and associated disorders. 2011;25:203-5.

16. Rains VS, Ditzler TF. Alcohol use disorders in cognitively impaired patients referred for geriatric assessment. Journal of Affective Disorders. 1993;12:55-64.

17. Saxton J, Munro CA, Butters MA, Schramke C, McNeil MA. Alcohol, dementia, and Alzheimer’s disease: comparison of neuropsychological profiles. Journal of Geriatric Psychiatry. 2000;13:141-9.

18. Woodburn KJ, Johnstone EC. Early-onset dementia in Lothian, Scotland: ananalys is of clinical features and patterns of decline. Health Bulletin. 1999;57:384-92.

19. Anttila T, Helkala EL, Viitanen M, Kareholt I, Fratiglioni L, Winblad B, et al. Alcohol drinking in middle age and subsequent risk of mild cognitive impairment and dementia in old age: a prospective population based study. BMJ. 2004;329:539. doi: 10.1136/bmj.38181.418958.BE.

20. Espeland MA, Gu L, Masaki KH, Langer RD, Coker LH, Stefanick ML, et al. Association between reported alcohol intake and cognition: results from the Women's Health Initiative Memory Study. American journal of epidemiology. 2005;161:228-38. doi: 10.1093/aje/kwi043.

21. Handing EP, Andel R, Kadlecova P, Gatz M, Pedersen NL. Midlife alcohol consumption and risk of dementia over 43 years of follow-up: a population-based study from the Swedish Twin Registry. The Journals of Gerontology Series A, Biological Sciences and Medical Sciences. 2015;70:1248-54.

22. Järvenpää T, Rinne JO, Koskenvuo M, Raiha I, Kaprio J. Binge drinking in midlife and dementia risk. Epidemiology. 2005;16:766-71.

23. Langballe EM, Ask H, Holmen J, Stordal E, Saltvedt I, Selbaek G, et al. Alcohol consumption and risk of dementia up to 27 years later in a large, population-based sample: the HUNT study, Norway. European journal of epidemiology. 2015;30:1049-56. doi: 10.1007/s10654-015-0029-2.

24. Mukamal KJ, Kuller LH, Fitzpatrick AL, Longstreth WTJ, Mittleman MA, Siscovick DS. Prospective study of alcohol consumption and risk of dementia in older adults. Journal of American Medical Association. 2003;289:1405-13.

25. Paganini-Hill A, Kawas CH, Corrada MM. Lifestyle factors and dementia in the oldest-old: the 90? study. Alzheimer disease and associated disorders. 2016;30:21-6.

26. Ruitenberg A, van Swieten JC, Witteman JCM, Mehta KM, van Duijn CM, Hofman A, et al. Alcohol consumption and risk of dementia: the Rotterdam Study. Lancet. 2002;359:281-6.

27. Truelsen T, Thudium D, Gronbaek M. Amount and type of alcohol and risk of dementia. Neurology. 2002;59:1313-9.

28. Zhou S, Zhou R, Zhong T, Li R, Tan J, Zhou H. Association of smoking and alcohol drinking with dementia risk among elderly men in China. Current Alzheimer Research. 2014;11:899-907.

29. Cervilla JA, Prince M, Mann A. Smoking, drinking, and incident cognitive impairment: a cohort community based study included in the gospel Oak project. Journal of neurology, neurosurgery, and psychiatry. 2000;68:622-6.

30. Huang W, Qiu C, Winblad B, Fratiglioni L. Alcohol consumption and incidence of dementia in a community sample aged 75 years and older. Journal of Clinical Epidemiology. 2002;55:959-64.

31. Luchsinger JA, Tang MX, Siddiqui M, Shea S, Mayeux R. Alcohol intake and risk of dementia. Journal of the American Geriatrics Society. 2004;52:540-6. doi: 10.1111/j.1532-5415.2004.52159.x.

32. Mehlig K, Skoog I, Guo X, Schutze M, Gustafson D, Waern M, et al. Alcoholic beverages and incidence of dementia: 34-year follow-up of the prospective population study of women in Goteborg. American journal of epidemiology. 2008;167:684-91. doi: 10.1093/aje/kwm366.

33. Simons LA, Simons J, McCallum J, Friedlander Y. Lifestyle factors and risk of dementia: Dubbo study of the elderly. Medical Journal of Australia. 2006;184:68-70.

34. Solfrizzi V, D'Introno A, Colacicco AM, Capurso C, Del Parigi A, Baldassarre G, et al. Alcohol consumption, mild cognitive impairment, and progression to dementia. Neurology. 2007;68:1790-9. doi: 10.1212/01.wnl.0000262035.87304.89.

35. Stampfer MJ, Kang JH, Chen J, Cherry R, Grodstein F. Effects of moderate alcohol consumption on cognitive function in women. New England Journal of Medicine. 2005;352:245-53.

36. Beulens JW, de Bruijne LM, Stolk RP, Peeters PH, Bots ML, Grobbee DE, et al. High dietary glycemic load and glycemic index increase risk of cardiovascular disease among middle-aged women: a population-based follow-up study. J Am Coll Cardiol. 2007;50:14-21. doi: 10.1016/j.jacc.2007.02.068.

37. Elwood P, Galante J, Pickering J, Palmer S, Bayer A, Ben-Shlomo Y, et al. Healthy lifestyles reduce the incidence of chronic diseases and dementia: evidence from the Caerphilly cohort study. PloS one. 2013;8:e81877. doi: 10.1371/journal.pone.0081877.

38. Emberson JR, Shaper AG, Wannamethee SG, Morris RW, Whincup PH. Alcohol intake in middle age and risk of cardiovascular disease and mortality: accounting for intake variation over time. American journal of epidemiology. 2005;161:856-63.

39. Englund U, Nordstrom P, Nilsson J, Hallmans G, Svensson O, Bergstrom U, et al. Active commuting reduces the risk of wrist fractures in middle-aged women-the UFO study. Osteoporosis International. 2013;24:533-40. doi: 10.1007/s00198-012-1988-8.

40. Flood A, Rastogi T, Wirfalt E, Mitrou PN, Reedy J, Subar AF, et al. Dietary patterns as identified by factor analysis and colorectal cancer among middle-aged Americans. The American journal of clinical nutrition. 2008;88:176-84.

41. Iso H, Baba S, Mannami T, Sasaki S, Okada K, Konishi M, et al. Alcohol consumption and risk of stroke among middle-aged men: the JPHC study cohort I. Stroke. 2004;35:1124-9.

42. Lin Y, Kikuchi S, Tamakoshi A, Wakai K, Kawamura T, Iso H, et al. Alcohol consumption and mortality among middle-aged and elderly Japanese men and women. Annals of Epidemiology. 2005;15:590-7. doi: 10.1016/j.annepidem.2004.10.010.

43. Moayyeri A, Kaptoge S, Luben RN, Wareham NJ, Bingham S, Reeve J, et al. Estimation of absolute fracture risk among middle-aged and older men and women: the EPIC-Norfolk population cohort study. European journal of epidemiology. 2009;24:259-66. doi: 10.1007/s10654-009-9337-8.

44. Ostbye T, Taylor DH, Jung SH. A longitudinal study of the effects of tobacco smoking and other modifiable risk factors on ill health in middle-aged and old Americans: results from the Health and Retirement Study and Asset and Health Dynamics among the Oldest Old survey. Preventive Medicine 2002;34:334-45.

45. Otani T, Iwasaki M, Yamamoto S, Sobue T, Hanaoka T, Inoue M, et al. Alcohol consumption, smoking, and subsequent risk of colorectal cancer in middle-aged and elderly Japanese men and women: Japan Public Health Center-based prospective study. Cancer epidemiology, biomarkers & prevention : a publication of the American Association for Cancer Research, cosponsored by the American Society of Preventive Oncology. 2003;12:1492-500.

46. Qiu D, Mei J, Tanihata T, Kawaminami K, Minowa M. A cohort study on Cerebrovascular Disease in middle-aged and elderly population in rural areas in Jiangxi Province, China. Journal of Epidemiology. 2003;13:149-56.

47. Sabia S, Nabi H, Kivimaki M, Shipley MJ, Marmot MG, Singh-Manoux A. Health behaviors from early to late midlife as predictors of cognitive function: The Whitehall II study. American journal of epidemiology. 2009;170:428-37. doi: 10.1093/aje/kwp161.

48. Sabia S, Gueguen A, Berr C, Berkman L, Ankri J, Goldberg M, et al. High alcohol consumption in middle-aged adults is associated with poorer cognitive performance only in the low socio-economic group. Results from the GAZEL cohort study. Addiction (Abingdon, England). 2011;106:93-101. doi: 10.1111/j.1360-0443.2010.03106.x.

49. Stevens RJ, Roddam AW, Spencer EA, Pirie KL, Reeves GK, Green J, et al. Factors associated with incident and fatal pancreatic cancer in a cohort of middle-aged women. International journal of cancer. 2009;124:2400-5. doi: 10.1002/ijc.24196.

50. Sun Q, Townsend MK, Okereke OI, Rimm EB, Hu FB, Stampfer MJ, et al. Alcohol consumption at midlife and successful ageing in women: a prospective cohort analysis in the nurses' health study. PLoS Medicine. 2011;8:e1001090. doi: 10.1371/journal.pmed.1001090. .

51. Tabak C, Smit HA, Rasanen L, Fidanza F, Menotti A, Nissinen A, et al. Alcohol consumption in relation to 20-year COPD mortality and pulmonary function in middle-aged men from three European countries. Epidemiology. 2001;12:239-45.

52. Virtaa JJ, Jarvenpaa T, Heikkila K, Perola M, Koskenvuo M, Raiha I, et al. Midlife alcohol consumption and later risk of cognitive impairment: a twin follow-up study. Journal of Alzheimer's disease. 2010;22:939-48. doi: 10.3233/jad-2010-100870.

53. Waki K, Noda M, Sasaki S, Matsumura Y, Takahashi Y, Isogawa A, et al. Alcohol consumption and other risk factors for self-reported diabetes among middle-aged Japanese: a population-based prospective study in the JPHC study cohort I. Diabetic medicine : a journal of the British Diabetic Association. 2005;22:323-31. doi: 10.1111/j.1464-5491.2004.01403.x.

54. Wang L, Lee IM, Manson JE, Buring JE, Sesso HD. Alcohol consumption, weight gain, and risk of becoming overweight in middle-aged and older women. Archives of Internal Medicine. 2010;170:453-61.

55. Wannamethee SG, Shaper AG. Taking up regular drinking in middle age: effect on major coronary heart disease events and mortality. Heart. 2002;87:32-6.

56. Wannamethee SG, Shaper AG. Alcohol, body weight, and weight gain in middle-aged men. American Journal of Clinical Nutrition. 2003;77:1312-7.

57. Willcox BJ, He Q, Chen R, Yano K, Masaki KH, Grove JS, et al. Midlife risk factors and healthy survival in men. Jama. 2006;296:2343-50. doi: 10.1001/jama.296.19.2343.

58. Xu Q, Anderson D, Courtney M. A longitudinal study of the relationship between lifestyle and mental health among midlife and older women in Australia: findings from the Healthy Aging of Women Study. Health care for women international. 2010;31:1082-96. doi: 10.1080/07399332.2010.486096.

59. Artero S, Ancelin ML, Portet F, Dupuy A, Berr C, Dartigues JF, et al. Risk profiles for mild cognitive impairment and progression to dementia are gender specific. Journal of Neurology, Neurosurgery, and Psychiatry. 2008;79:979-84. doi: 10.1136/jnnp.2007.136903.

60. Fellows L, Bergman H, Wolfson C, Chertkow H. Can clinical data predict progression to dementia in amnestic mild cognitive impairment? Can J Neurol Sci. 2008;35:314-22.

61. Li J, Wang YJ, Zhang M, Xu ZQ, Gao CY, Fang CQ, et al. Vascular risk factors promote conversion from mild cognitive impairment to Alzheimer disease. Neurology. 2011;76:1485-91. doi: 10.1212/WNL.0b013e318217e7a4.

62. Luck T, Riedel-Heller SG, Luppa M, Wiese B, Bachmann C, Jessen F, et al. A hierarchy of predictors for dementia-free survival in old-age: results of the AgeCoDe study. Acta Psychiatr Scand Suppl. 2014;129:63-72. doi: 10.1111/acps.12129.

63. Velayudhan L, Poppe M, Archer N, Proitsi P, Brown RG, Lovestone S. Risk of developing dementia in people with diabetes and mild cognitive impairment. The British journal of psychiatry : the journal of mental science. 2010;196:36-40. doi: 10.1192/bjp.bp.109.067942.

64. Xu G, Liu X, Yin Q, Zhu W, Zhang R, Fan X. Alcohol consumption and transition of mild cognitive impairment to dementia. Psychiatry and Clinical Neurosciences. 2009;63:43-9.

65. Antsey KJ, Mack HA, Cherbuin N. Alcohol consumption as a risk factor for dementia and cognitive decline: meta-analysis of prospective studies. American Journal of Geriatric Psychiatry. 2009;17:542-55.

66. Piazza-Gardner AK, Faffud TJ, Barry AE. The impact of alcohol on Alzheimer's disease: a systematic review. Aging and Mental Health. 2013;17:133-46. doi: 10.1080/13607863.2012.742488.

67. Crego A, Holguin SR, Parada M, Mota N, Corral M, Cadaveira F. Binge drinking affects attention and visual working memory processing in young university students. Alcoholism: Clinical & Experimental Research. 2009;33:1-10. doi: 10.1111/j.1530-0277.2009.01025.x.

68. Scaife JC, Duka T. Behavioural measures of frontal lobe function in a population of young drinkers with binge drinking pattern. Pharmacology, Biochemistry and Behavior. 2009;93:354-62. doi: 10.1016/j.pbb.2009.05.015.

69. Townshend JM, Duka T. Binge drinking, cognitive performance and mood in a population of young social drinkers. Alcoholism: Clinical & Experimental Research. 2005;29:317-25.

70. Brayne C, Gill C, Huppert FA, Barkley C, Gehlhaar E, Girling DM, et al. Vascular risks and incident dementia: results from a cohort study of the very old. Dementia and Geriatric Cognitive Disorders. 1998;9:175-80.

71. Broe GA, Henderson AS, Creasey H, McCusker E, Korten AE, Jorm AF, et al. A case-control study of Alzheimer's disease in Australia. Neurology. 1990;40:1698-707.

72. Dai Q, Borenstein AR, Wu Y, Jackson JC, Larson EB. Fruit and vegetable juices and Alzheimer’s disease: the Kame project. American Journal of Medicine. 2006;119:751-9. doi: 10.1016/j.amjmed.2006.03.045.

73. Deng J, Zhou DH, Li J, Wang YJ, Gao C, Chen M. A 2-year follow-up study of alcohol consumption and risk of dementia. Clinical Neurology and Neurosurgery. 2006;108:378-83.

74. Ferini-Strambi L, Smirne S, Garancini P, Pinto P, Franceschi M. Clinical and epidemiological aspects of Alzheimer's disease with presenile onset: a case control study. Neuroepidemiology. 1990;9:39-49.

75. Fratiglioni L, Ahlbom A, Viitanen M, Winblad B. Risk factors for late-onset Alzheimer's disease: a population-based, case-control study. Annals of Neurology. 1993;33:258-66.

76. Garcia AM, Ramon-Bou N, Porta M. Isolated and joint effects of tobacco and alcohol consumption on risk of Alzheimer's disease. Journal of Alzheimer's Disease. 2010;20:577-86.

77. Graves AB, White E, Koepsell TD, Reifler BV, van Belle G, Larson EB, et al. A case-control study of Alzheimer's disease. Annals of Neurology. 1990;28:766-74.

78. Harmancini H, Emre M, Gurvit H, Bilgic B, Hanagasi H, Gurol E, et al. Risk factors for Alzheimer disease: a population-based case-control study in Istanbul, Turkey. Alzheimer Disease and Associated Disorders. 2003;17:139-45.

79. Harwood DG, Barker WW, Loewenstein DA, Ownby RL, St George-Hyslop P, Mullan M, et al. A cross-ethnic analysis of risk factors for AD in white Hispanics and white non-Hispanics. Neurology. 1999;52:551-6.

80. Hebert LE, Scherr PA, Beckett LA, Funkenstein HH, Albert MS, Chown MJ, et al. Relation of smoking and alcohol consumption to incident Alzheimer's disease. American journal of epidemiology. 1992;135:347-55.

81. Kokmen E, Beard CM, Chandra V, Offord KP, Schoenberg BS, Ballard DJ. Clinical risk factors for Alzheimer's disease: a population-based case-control study. Neurology. 1991;41.

82. Kondo K, Niino M, Shido K. A case-control study of Alzheimer's disease in Japan--significance of life-styles. Dementia 1994;5:314-26.

83. Kukull WA, Larson EB, Bowen JD, McCormick WC, Teri L, Pfanschmidt ML, et al. Solvent exposure as a risk factor for Alzheimer's disease: a case-control study. American journal of epidemiology. 1995;141:1059-71; discussion 72-9.

84. Leibovici D, Ritchie K, Ledesert B, Touchon J. The effects of wine and tobacco consumption on cognitive performance in the elderly: a longitudinal study of relative risk. International Journal of Epidemiology. 1999;28:77-81.

85. Li G, Shen YC, Li YT, Chen CH, Zhau YW, Silverman JM. A case-control study of Alzheimer's disease in China. Neurology. 1992;42:1481-8.

86. Lindsay J, Laurin D, Verreault R, Hebert R, Helliwell B, Hill G, et al. Risk factors for Alzheimer's disease: a prospective analysis of the Canadian study of health and aging. American journal of epidemiology. 2002;156:445-53.

87. Mendez MF, Underwood KL, Zander BA, Mastri AR, Sung JH, Frey WH. Risk factors in Alzheimer's disease: a clinicopathologic study. Neurology. 1992;42:770-5.

88. Tyas SL, Manfreda J, Strain LA, Montgomery PR. Risk factors for Alzheimer's disease: a population-based, longitudinal study in Manitoba, Canada. International Journal of Epidemiology. 2001;30:590-7.

89. Wang PN, Wang SJ, Hong CJ, Liu TT, Fuh JL, Chi CW, et al. Risk factors for Alzheimer's disease: a case-control study. Neuroepidemiology. 1997;16:234-40.

90. Gureje O, Ogunniyi A, Kola L, Abiona T. Incidence of and risk factors for dementia in the Ibadan study of aging. Journal of the American Geriatrics Society. 2011;59:869-74. doi: 10.1111/j.1532-5415.2011.03374.x.

91. Yip AG, Brayne C, Matthews FE. Risk factors for incident dementia in England and Wales: the Medical Research Council Cognitive Function and Ageing Study. A population-based nested case-control study. Age Ageing. 2006;35:154-60.

92. Neafsey EJ, Collins MA. Moderate alcohol consumption and cognitive risk. Neuropsychiatr Dis Treat. 2011;7:465-84. doi: 10.2147/NDT.S23159.

93. Au Yeung SL, Jiang C, Zhang W, Lam TH, Cheng KK, Leung GM, et al. Moderate alcohol use and cognitive function in the Guangzhou Biobank cohort study. Annals of Epidemiology. 2010;20:873-82.

94. Bond GE, Burr R, McCurry SM, Graves AB, Larson EB. Alcohol, aging, and cognitive performance in a cohort of Japanese Americans aged 65 and older: the Kame project. International Psychogeriatrics. 2001;13:207-23.

95. Bond GE, Burr R, McCurry SM, Rice MM, Borenstein AR, Kukull WA, et al. Alcohol, gender, and cognitive performance: a longitudinal study comparing older Japanese and non-Hispanic white Americans. Journal of Aging and Health. 2004;16:615-40.

96. Bond GE, Burr RL, McCurry SM, Rice MM, Borenstein AR, Larson EB. Alcohol and cognitive performance: a longitudinal study of older Japanese Americans. The Kame project. International Psychogeriatrics. 2005;17:653-68.

97. Britton A, Singh-Manoux A, Marmot M. Alcohol consumption and cognitive function in the Whitehall II study. American journal of epidemiology. 2004;160:240-7.

98. Broe GA, Creasey H, Jorm AF, Bennett HP, Casey B, Waite LM, et al. Health habits and risk of cognitive impairment and dementia in old age: a prospective study on the effects of exercise, smoking and alcohol consumption. Australian and New Zealand Journal of Public Health. 1998;22:621-3.

99. Carmelli D, Swan GE, Reed T, Schellenberg GD, Christian JC. The effect of apolipoprotein E epsilon4 in the relationships of smoking and drinking to cognitive function. Neuroepidemiology. 1999;18:125-33.

100. Chan KK, Chiu KC, Chu LW. Association between alcohol consumption and cognitive impairment in Southern Chinese older adults. International Journal of Geriatric Psychiatry. 2010;25:1272-9.

101. Corley J, Jia X, Brett CE, Gow AJ, Starr JM, Kyle JA, et al. Alcohol intake and cognitive abilities in old age: the Lothian birth cohort 1936 study. Neuropsychology 2011;25:166-75.

102. Dufouil C, Ducimetiere P, Alperovitch A. Sex differences in the association between alcohol consumption and cognitive performance. EVA study group. Epidemiology of vascular aging. American journal of epidemiology. 1997;146:405-12.

103. Edelstein SL, Kritz-Silverstein D, Barrett-Connor E. Prospective association of smoking and alcohol use with cognitive function in an elderly cohort. Journal of Women's Health. 1998;7:1271-81.

104. Elias PK, Elias MF, D'Agostino RB, Silbershatz H, Wolf PA. Alcohol consumption and cognitive performance in the Framingham heart study. American journal of epidemiology. 1999;150:580-9.

105. Ganguli M, Vander Bilt J, Saxton JA, Shen C, Dodge HH. Alcohol consumption and cognitive function in late life: a longitudinal community study. Neurology. 2005;65:1210-7.

106. Gross AL, Rebok GW, Ford DE, Chu AY, Gallo JJ, Liang KY, et al. Alcohol consumption and domain-specific cognitive function in older adults: longitudinal data from the Johns Hopkins precursors study. The Journals of Gerontology. 2011;66:39-47.

107. Hendrie HC, Gao S, Hall KS, Hui SL, Unverzagt FW. The relationship between alcohol consumption, cognitive performance, and daily functioning in an urban sample of older black Americans. Journal of the American Geriatrics Society. 1996;44:1158-65.

108. Kalmijn S, van Boxtel MP, Verschuren MW, Jolles J, Launer LJ. Cigarette smoking and alcohol consumption in relation to cognitive performance in middle age. American journal of epidemiology. 2002;156:936-44.

109. Krahn D, Freese J, Hauser R, Barry K, Goodman B. Alcohol use and cognition at mid-life: the importance of adjusting for baseline cognitive ability and educational attainment. Alcoholism: Clinical & Experimental Research. 2003;27:1162-6.

110. Leroi I, Sheppard JM, Lyketsos CG. Cognitive function after 11.5 years of alcohol use: relation to alcohol use. American journal of epidemiology. 2002;156:747-52.

111. Ngandu T, Helkala EL, Soininen H, Winblad B, Tuomilehto J, Nissinen A, et al. Alcohol drinking and cognitive functions: findings from the cardiovascular risk factors aging and dementia (CAIDE) study. Dementia and Geriatric Cognitive Disorders. 2007;23:140-9.

112. Reid MC, Van Ness PH, Hawkins KA, Towle V, Concato J, Guo Z. Light to moderate alcohol consumption is associated with better cognitive function among older male veterans receiving primary care. Journal of Geriatric Psychiatry. 2006;19:98-105.

113. Richards M, Hardy R, Wadsworth ME. Alcohol consumption and midlife cognitive change in the British 1946 birth cohort study. Alcohol and Alcoholism. 2005;40:112-7.

114. Rodgers B, Windsor TD, Anstey KJ, Dear KB, FJ A, Christensen H. Non-linear relationships between cognitive function and alcohol consumption in young, middle-aged and older adults: the PATH through life project. Addiction (Abingdon, England). 2005;100:1280-90.

115. Wright CB, Elkind MS, Luo X, Paik MC, Sacco RL. Reported alcohol consumption and cognitive decline: the northern Manhattan study. Neuroepidemiology. 2006;27:201-7.

116. Zanjani F, Downer BG, Kruger TM, Willis SL, Schaie KW. Alcohol effects on cognitive change in middle-aged and older adults. Aging and Mental Health. 2013;17:12-23.

117. Zhou H, Deng J, Li J, Wang Y, Zhang M, He H. Study of the relationship between cigarette smoking, alcohol drinking and cognitive impairment among elderly people in China. Age Ageing. 2003;32:205-10.

118. Zuccala G, Onder G, Pedone C, Cesari M, Landi F, Bernabei R, et al. Dose-related impact of alcohol consumption on cognitive function in advanced age: results of a multicenter survey. Alcoholism: Clinical & Experimental Research. 2001;25:1743-8.

119. Kivipelto M, Rovio S, Ngandu T, Kareholt I, Eskelinen M, Winblad B, et al. Apolipoprotein E epsilon4 magnifies lifestyle risks for dementia: A population-based study. Journal of Cellular and Molecular Medicine. 2008;12:2762-71.

120. Larrieu S, Letenneur L, Helmer C, Dartigues JF, Barberger-Gateau P. Nutritional factors and risk of incident dementia in the PAQUID longitudinal cohort. The Journal of Nutrition Health and Aging. 2004;8:150-4.

121. Orgogozo JM, Dartigues JF, Lafont S, Letenneur L, Commenges D, Salamon R, et al. Wine consumption and dementia in the elderly: A prospective community study in the Bordeaux area. Revue de neuropsychologie. 1997;153:185-92.

122. Peters R, Beckett N, Geneva M, Tzekova M, Lu FH, Poulter R, et al. Sociodemographic and lifestyle risk factors for incident dementia and cognitive decline in the HYVET. Age Ageing. 2009;38:521-7.

123. Crego A, Hoguin SR, Parada M, Mota N, Corral M, Cadaveira F. Reduced anterior prefrontal cortex activation in young binge drinking during a visual working memory task. Drug and Alcohol Dependence. 2010;109:45-56.

124. Goudriaan AE, Grekin ER, Sher KJ. Goudriaan AE, Grekin ER, Sher KJ. Decision making and binge drinking: a longitudinal study. Alcoholism: Clinical & Experimental Research. 2007;31:928-38.

125. Hartley DE, Elsabagh S, File SE. Binge drinking and sex: effects on mood and cognitive function in healthy young volunteers. Pharmacology Biochemistry & Behavior. 2004;78:611-9.

126. Heffernan T, Clark R, Bartholomew J, Ling J, Stephens S. Does binge drinking in teenagers affect their everyday prospective memory? . Drug and Alcohol Dependence. 2010;109:73-8.

127. Heffernan T, O'Neill T. Time based prospective memory deficits associated with binge drinking: evidence from the Cambridge Prospective Memory Test (camprompt). Drug and Alcohol Dependence. 2012;123:207-12.

128. Johnson CA, Xiao L, Palmer P, Sun P, Wang Q, Wei Y, et al. Affective decision-making deficits, linked to a dysfunctional ventromedial prefrontal cortex, revealed in 10th grade Chinese adolescent binge drinking. Neuropsychologia. 2008;46:714-26.

129. Nederkoorn C, Baltus M, Guerrieri R, Wiers RW. Heavy drinking is associated with deficient response inhibition in women but not in men. Pharmacology, Biochemistry & Behavior. 2009;93:331-6.

130. Parada M, Corral M, Caamaño-Isorna F, Mota N, Crego A, Holguin SR, et al. Binge drinking and declarative memory in university students. Alcoholism: Clinical & Experimental Research. 2011;35:1475-84.

131. Parada M, Corral M, Mota N, Crego A, Rodríguez Holguín S, Cadaveira F. Executive functioning and alcohol binge drinking in university students. Addictive Behaviors. 2012;37:167-72.

132. Sanhueza C, García-Moreno LM, Expósito J. Weekend alcoholism in youth and neurocognitive aging. Psicothema. 2011;23:209-14.

133. Schweinsburg AD, McQueeny T, Nagel BJ, Eyler LT, Tapert SF. A preliminary study of functional magnetic resonance imaging response during verbal encoding among adolescent binge drinking. Alcohol 2010;44:111-7.

134. Schweinsburg AD, Schweinsburg BC, Nagel BJ, Eyler LT, Tapert SF. Neural correlates of verbal learning in adolescent alcohol and marijuana users. Addiction (Abingdon, England). 2011;106:564-73. doi: 10.1111/j.1360-0443.2010.03197.x.

135. Squeglia LM, Schweinsburg AD, Pulido C, Tapert SF. Adolescent binge drinking linked to abnormal spatial working memory brain activation: differential gender effects. Alcoholism: Clinical & Experimental Research. 2011;35:1831-41.

136. Squeglia LM, Sorg SF, Schweinsburg AD, Wetherill RR, Pulido C, Tapert SF. Binge drinking differentially affects adolescent male and female brain morphometry. Psychopharmacology. 2012;220:529-39.

137. Weissenborn R, Duka T. Acute alcohol effects on cognitive function in social drinkers: their relationship to drinking habits. Psychopharmacology. 2003;165:306-12.

138. Xiao L, Bechara A, Grenard LJ, Stacy WA, Palmer P, Wei Y, et al. Affective decision-making predictive of Chinese adolescent drinking behaviors. Journal of International Neuropsychological Society. 2009;15:547-57.

139. Bachman DL, Green RC, Benke KS, Cupples LA, Farrer LA. Comparison of Alzheimer’s disease risk factors in white and African American families. Neurology. 2003;60:1372-4.

140. Harwood DG, Kalechstein A, Barker WW, Strauman S, St George-Hyslop P, Iglesias C, et al. The effect of alcohol and tobacco consumption, and apolipoprotein E genotype, on the age of onset in Alzheimer's disease. International Journal of Geriatric Psychiatry. 2010;25:511-8. doi: 10.1002/gps.2372.

141. Ogunniyi A, Hall KS, Gureje O, Baiyewu O, Gao S, Unverzagt FW, et al. Risk factors for incident Alzheimer's disease in African Americans and Yoruba. Metab Brain Dis. 2006;21:235-40. doi: 10.1007/s11011-006-9017-2.

142. Rosen J, Colantonio A, Becker JT, Lopez OL, DeKosky ST, Moss HB. Effects of a history of heavy alcohol consumption on Alzheimer’s disease. British Journal of Psychiatry. 1993;163:358-863.

143. Tsolaki M, Fountoulakis K, Chantzi E, Kazis A. Risk factors for clinically diagnosed Alzheimer’s disease: A case-control study of a Greek population. International Psychogeriatrics. 1997;9:327-41.

144. Tyas SL, Koval JJ, Pederson LL. Does an interaction between smoking and drinking influence the risk of Alzheimer’s disease? Results from three Canadian data sets. Statistics in Medicine. 2000;19:1685-96.

145. Yoshitake T, Kiyohara Y, Kato I, Ohmura T, Iwamoto H, Nakayama K, et al. Incidence and risk factors of vascular dementia and Alzheimer's disease in a defined elderly Japanese population: the Hisayama Study. Neurology. 1995;45:1161-8.

146. Heffernan TM, Ling TM, Bartholomew J. Self-rated prospective memory and central executive impairments in excessive alcohol users. Irish Journal of Psychological Medicine. 2004;21:122-4.

147. Piechatzek M, Indlekofer F, Daamen M, Glasmacher C, Lieb R, Pfister H, et al. Is moderate substance use associated with altered executive functioning in a population-based sample of young adults? Human Psychopharmacology. 2009;24:650-65. doi: 10.1002/hup.1069.

148. Townshend JM, Duka T. Attentional bias associated with alcohol cues: differences between heavy and occasional social drinkers. Psychopharmacology. 2001;157:67-74.

149. Amaducci LA, Fratiglioni L, Rocca WA, Fieschi C, Livrea P, Pedone D, et al. Risk factors for clinically diagnosed Alzheimer's disease: a case-control study of an Italian population. Neurology. 1986;36:922-31.

150. Au Yeung SL, Leung GM, Chan WM, Hui YF, Lam TH, Schooling CM. Moderate alcohol use and cognitive function in an elderly Chinese cohort. Journal of the American Geriatrics Society. 2011;59:172-4. doi: 10.1111/j.1532-5415.2010.03195.x.

151. Barnes DE, Covinsky KE, Whitmer RA, Kuller LH, Lopez OL, Yaffe K. Predicting risk of dementia in older adults: The late-life dementia risk index. Neurology. 2009;73:173-9. doi: 10.1212/WNL.0b013e3181a81636.

152. Cherbuin N, Reglade-Meslin C, Kumar R, Jacomb P, Easteal S, Christensen H, et al. Risk factors of transition from normal cognition to mild cognitive disorder: the PATH through Life Study. Dementia and Geriatric Cognitive Disorders. 2009;28:47-55. doi: 10.1159/000229025.

153. Commenges D, Scotet V, Renaud S, Jacqmin-Gadda H, Barberger-Gateau P, Dartigues JF. Intake of flavonoids and risk of dementia. European journal of epidemiology. 2000;16:357-63.

154. Dai Q, Pruett SB. Different effects of acute and chronic ethanol on LPS-induced cytokine production and TLR4 receptor behavior in mouse peritoneal macrophages. Journal of Immunotoxicology. 2006;3:217-25.

155. DeCarli C, Miller BL, Swan GE, Reed T, Wolf PA, Carmelli D. Cerebrovascular and brain morphologic correlates of mild cognitive impairment in the National Heart, Lung, and Blood Institute Twin Study. Arch Neurol. 2001;58:643-7.

156. Ensrud KE, Nevitt MC, Yunis C, Cauley JA, Seeley DG, Fox KM, et al. Correlates of impaired function in older women. Journal of the American Geriatrics Society. 1994;42:481-9.

157. French LR, Schuman LM, Mortimer JA, Hutton JT, Boatman RA, Christians B. A case-control study of dementia of the Alzheimer type. American journal of epidemiology. 1985;121:414-21.

158. Fujishima M, Kiyohara Y. Incidence and risk factors of dementia in a defined elderly Japanese population: the Hisayama study. Annals of the New York Academy of Sciences. 2002;977:1-8.

159. Galanis DJ, Joseph C, Masaki KH, Petrovitch H, Ross GW, White L. A longitudinal study of drinking and cognitive performance in elderly Japanese American men: the Honolulu-Asia Aging Study. Am J Public Health. 2000;90:1254-9.

160. Guralnik JM, Kaplan GA. Predictors of healthy aging: prospective evidence from the Alameda County study. Am J Public Health. 1989;79:703-8.

161. Heyman A, Wilkinson WE, Stafford JA, Helms MJ, Sigmon AH, Weinberg T. Alzheimer's disease: a study of epidemiological aspects. Annals of Neurology. 1984;15:335-41. doi: 10.1002/ana.410150406.

162. Hofman A, van Duijn CM. Alzheimer’s disease, Parkinson’s disease and smoking. Neurobiol Aging. 1990;11:295.

163. Iliffe S, Haines A, Booroff A, Goldenberg E, Morgan P, Gallivan S. Alcohol consumption by elderly people: a general practice survey. Age Ageing. 1991;20:120-3.

164. Kim KW, Park JH, Kim MH, Kim MD, Kim BJ, Kim SK, et al. A nationwide survey on the prevalence of dementia and mild cognitive impairment in South Korea. Journal of Alzheimer's Disease. 2011;23:281-91. doi: 10.3233/jad-2010-101221.

165. Launer LJ, Feskens EJ, Kalmijn S, Kromhout D. Smoking, drinking, and thinking. The Zutphen Elderly Study. American journal of epidemiology. 1996;143:219-27.

166. Lobo E, Dufouil C, Marcos G, Quetglas B, Saz P, Guallar E, et al. Is there an association between low-to-moderate alcohol consumption and risk of cognitive decline? American journal of epidemiology. 2010;172:708-16. doi: 10.1093/aje/kwq187.

167. Lopes MA, Furtado EF, Ferrioli E, Litvoc J, Bottino CM. Prevalence of alcohol-related problems in an elderly population and their association with cognitive impairment and dementia. Alcoholism: Clinical & Experimental Research. 2010;34:726-33. doi: 10.1111/j.1530-0277.2009.01142.x.

168. Luck T, Luppa M, Briel S, Matschinger H, Konig HH, Bleich S, et al. Mild cognitive impairment: incidence and risk factors: results of the leipzig longitudinal study of the aged. Journal of the American Geriatrics Society. 2010;58:1903-10. doi: 10.1111/j.1532-5415.2010.03066.x.

169. McGuire LC, Ajani UA, Ford ES. Cognitive functioning in late life: the impact of moderate alcohol consumption. Annals of Epidemiology. 2007;17:93-9.

170. Mukamal KJ, Chiuve SE, Rimm EB. Alcohol consumption and risk for coronary heart disease in men with healthy lifestyles. Archives of Internal Medicine. 2006;166:2145-50.

171. Rozzini R, Trabucchi M. Re: “Association between reported alcohol intake and cognition: results from the Women’s Health Initiative Memory Study”. American journal of epidemiology. 2005;162:294-5.

172. Rusanen M, Kivipelto M, Quesenberry CP, Jr., Zhou J, Whitmer RA. Heavy smoking in midlife and long-term risk of Alzheimer disease and vascular dementia. Archives of Internal Medicine. 2011;171:333-9. doi: 10.1001/archinternmed.2010.393.

173. Smith K, Flicker L, Dwyer A, Atkinson D, Almeida OP, Lautenschlager NT, et al. Factors associated with dementia in Aboriginal Australians. Aust N Z J Psychiatry. 2010;44:888-93. doi: 10.3109/00048674.2010.491816.

174. Tanaka N, Asada T, Kinoshita T, Yamashita F, Uno M. Alcohol consumption and risk of dementia. Lancet. 2002;360:491. doi: 10.1016/s0140-6736(02)09655-1.

175. Umegaki H, Iimuro S, Kaneko T, Araki A, Sakurai T, Ohashi Y, et al. Factors associated with lower Mini Mental State Examination scores in elderly Japanese diabetes mellitus patients. Neurobiology of Aging. 2008;29:1022-6. doi: 10.1016/j.neurobiolaging.2007.02.004.

176. Vincze G, Almos P, Boda K, Dome P, Bodi N, Szlavik G, et al. Risk factors of cognitive decline in residential care in Hungary. International Journal of Geriatric Psychiatry. 2007;22:1208-16. doi: 10.1002/gps.1815.

177. Wadley VG, McClure LA, Howard VJ, Unverzagt FW, Go RC, Moy CS, et al. Cognitive status, stroke symptom reports, and modifiable risk factors among individuals with no diagnosis of stroke or transient ischemic attack in the REasons for Geographic and Racial Differences in Stroke (REGARDS) Study. Stroke. 2007;38:1143-7. doi: 10.1161/01.str.0000259676.75552.38.

178. Wright CB, Elkind MS, Rundek T, Boden-Albala B, Paik MC, Sacco RL. Alcohol intake, carotid plaque, and cognition: the Northern Manhattan Study. Stroke. 2006;37:1160-4. doi: 10.1161/01.STR.0000217439.73041.b4.

179. Yaffe K, Fiocco AJ, Lindquist K, Vittinghoff E, Simonsick EM, Newman AB, et al. Predictors of maintaining cognitive function in older adults: the Health ABC study. Neurology. 2009;72:2029-35. doi: 10.1212/WNL.0b013e3181a92c36.

180. Yen CH, Yeh CJ, Wang CC, Liao WC, Chen SC, Chen CC, et al. Determinants of cognitive impairment over time among the elderly in Taiwan: results of the national longitudinal study. Archives of Gerontology and Geriatrics. 2010;50 Suppl 1:S53-7. doi: 10.1016/s0167-4943(10)70014-5.

181. Dufouil C, Tzourio C, Brayne C, Berr C, Amouyel P, Alperovitch A. Influence of apolipoprotein E genotype on the risk of cognitive deterioration in moderate drinkers and smokers. Epidemiology. 2000;11:280-4.

182. Shin IS, Stewart R, Kim JM, Kim SW, Yang SJ, Shin HY, et al. Mitochondrial aldehyde dehydrogenase polymorphism is not associated with incidence of Alzheimer's disease. International Journal of Geriatric Psychiatry. 2005;20:1075-80. doi: 10.1002/gps.1401.

183. Scarmeas N, Stern Y, Tang MX, Mayeaux R, Luchsinger JA. Mediterranean diet and risk for Alzheimer’s disease. Annals of Neurology. 2006;59:912-21. doi: 10.1002/ana.20854.

184. Anstey KJ, Jorm AF, Reglade-Meslin C, Maller J, Kumar R, von Sanden C, et al. Weekly alcohol consumption, brain atrophy, and white matter hyperintensities in a community-based sample aged 60 to 64 years. Psychosomatic Medicine. 2006;68:778-85. doi: 10.1097/01.psy.0000237779.56500.af.

185. de Bruin EA, Hulshoff Pol HE, Schnack HG, Janssen J, Bijl S, Evans AC, et al. Focal brain matter differences associated with lifetime alcohol intake and visual attention in male but not in female non-alcohol-dependent drinkers. NeuroImage. 2005;26:536-45. doi: 10.1016/j.neuroimage.2005.01.036.

186. den Heijer T, Vermeer SE, van Dijk EJ, Prins ND, Koudstaal PJ, van Duijn CM, et al. Alcohol intake in relation to brain magnetic resonance imaging findings in older persons without dementia. The American journal of clinical nutrition. 2004;80:992-7.

187. Ding J, Eigenbrodt ML, Mosley TH, Jr., Hutchinson RG, Folsom AR, Harris TB, et al. Alcohol intake and cerebral abnormalities on magnetic resonance imaging in a community-based population of middle-aged adults: the Atherosclerosis Risk in Communities (ARIC) study. Stroke. 2004;35:16-21. doi: 10.1161/01.str.0000105929.88691.8e.

188. Kubota M, Nakazaki S, Hirai S, Saeki N, Yamaura A, Kusaka T. Alcohol consumption and frontal lobe shrinkage: study of 1432 nonalcoholic subjects. Journal of Neurology, Neurosurgery, and Psychiatry. 2001;71:104-6.

189. Mukamal KJ, Longstreth WT, Jr., Mittleman MA, Crum RM, Siscovick DS. Alcohol consumption and subclinical findings on magnetic resonance imaging of the brain in older adults: the cardiovascular health study. Stroke. 2001;32:1939-46.

190. Taki Y, Goto R, Evans A, Zijdenbos A, Neelin P, Lerch J, et al. Voxel-based morphometry of human brain with age and cerebrovascular risk factors. Neurobiology of Aging. 2004;25:455-63. doi: 10.1016/j.neurobiolaging.2003.09.002.

191. Lindeman RD, Wayne S, Baumgartner RN, Garry PJ. Cognitive function in drinkers compared to abstainers in the new mexico elder health survey. The Journals of Gerontology Series A, Biological sciences and medical sciences. 2005;60:1065-70.

192. Schinka JA, Vanderploeg RD, Rogish M, Ordorica PA. Effects of alcohol and cigarette use on cognition in middle-aged adults. Journal of International Neuropsychological Society. 2002;8:683-90.

193. Zhang Y, Heeren T, Curtis Ellison R. Education modifies the effect of alcohol on memory impairment: the Third National Health and Nutrition Examination Survey. Neuroepidemiology. 2005;24:63-9.

194. Cervilla JA, Prince M, Joels S, Lovestone S, Mann A. Long-term predictors of cognitive outcome in a cohort of older people with hypertension. The British journal of psychiatry : the journal of mental science. 2000;177:66-71.

195. Hébert R, Lindsay J, Verreault R, Rockwood K, Hill G, Dubois MF. Vascular dementia : incidence and risk factors in the Canadian study of health and aging. Stroke. 2000;31:1487-93.

196. Kivipelto M, Helkala EL, Laakso MP, Hanninen T, Hallikainen M, Alhainen K, et al. Midlife vascular risk factors and Alzheimer's disease in later life: longitudinal, population based study. BMJ. 2001;322:1447-51.

197. Verhaeghen P, Borchelt M, Smith J. Relation between cardiovascular and metabolic disease and cognition in very old age: cross-sectional and longitudinal findings from the Berlin aging study. Health Psychology. 2003;22:559-69.

198. Luchsinger JA, Mayeux R. Dietary factors and Alzheimer's disease. Lancet Neurology. 2004;3:579-87.

199. Canadian Study of Health and Aging. The Canadian study of health and aging: Risk factors for Alzheimer's disease in Canada. Neurology. 1994;44:2073-80.

200. Cattin L, Bordin P, Fonda M, Adamo C, Barbone F, Bovenzi M, et al. Factors associated with cognitive impairment among older Italian inpatients. Journal of the American Geriatrics Society. 1997;45:1324-30.

201. Colsher PL, Wallace RB. Elderly men with histories of heavy drinking: correlates and consequences. J Stud Alcohol. 1990;51:528-35.

202. Christian JC, Reed T, Carmelli D, Page WF, Norton JA, Jr., Breitner JC. Self-reported alcohol intake and cognition in aging twins. J Stud Alc. 1995;56:414-6.

203. Dent OF, Sulway MR, Broe GA, Creasey H, Kos SC, Jorm AF, et al. Alcohol consumption and cognitive performance in a random sample of Australian soldiers who served in the second world war. BMJ. 1997;314:1655-7.

204. Geroldi C, Rozzini R, Frisoni GB, Trabucchi M. Assessment of alcohol consumption and alcoholism in the elderly. Alcohol. 1994;11:513-6.

205. Goodwin JS, Sanchez CJ, Thomas P, Hunt C, Garry PJ, Goodwin JM. Alcohol intake in a healthy elderly population. Am J Public Health. 1987;77:173-7.

206. Gorelick PB, Brody J, Cohen D, Freels S, Levy P, Doliear W, et al. Risk factors for dementia associated with multiple cerebral infarcts. A case-control analysis in predominantly African-American hospital-based patients. Arch Neurol. 1993;50:714-20.

207. Hebert LE, Scherr PA, Beckett LA, Albert MS, Rosner B, Taylor JO, et al. Relation of smoking and low-to-moderate alcohol consumption to change in cognitive function: A longitudinal study in a defined community of older persons. American journal of epidemiology. 1993;137:881-91.

208. Lammi UK, Kivela SL, Nissinen A, Punsar S, Puska P, Karvonen M. Mental disability among elderly men in Finland: Prevalence, predictors, and correlates. Acta Psychiatr Scand Suppl. 1989;80:459-68.

209. Letenneur L, Dartigues JF, Orgogozo JM. Wine consumption in the elderly. Annals of Internal Medicine. 1993;118:317-8.

210. Lindsay J, Hebert R, Rockwood K. The Canadian Study of Health and Aging: risk factors for vascular dementia. Stroke. 1997;28:526-30.

211. Mangione CM, Seddon JM, Cook EF, Krug JH, Jr., Sahagian CR, Campion EW, et al. Correlates of cognitive function scores in elderly outpatients. Journal of the American Geriatrics Society. 1993;41:491-7.

212. Mellström D, Rundgren A, Svanborg A. Previous alcohol consumption and its consequences of ageing, morbidity and mortality in men aged 70-75. Age and Ageing. 1981;10:277-86.

213. Saunders PA, Copeland JR, Dewey ME, Davidson IA, McWilliam C, Sharma V, et al. Heavy drinking as a risk factor for depression and dementia in elderly men. Findings from the Liverpool longitudinal community study. British Journal of Psychiatry. 1991;159:213-6.

214. Scherr PA, Albert MS, Funkenstein HH, Cook NR, Hennekens CH, Branch LG, et al. Correlates of cognitive function in an elderly community population. American journal of epidemiology. 1988;128:1084-101.

215. Teri L, Hughes JP, Larson EB. Cognitive deterioration in Alzheimer's disease: behavioral and health factors. Journal of gerontology. 1990;45:P58-63.

216. Ueda K, Kawano H, Hasuo Y, Fujishima M. Prevalence and etiology of dementia in a Japanese community. Stroke. 1992;23:798-803.
